# Biopsy‐based single‐cell transcriptomics reveals MAIT cells as potential targets for controlling fibrosis‐related liver inflammation due to chronic hepatitis‐B infection

**DOI:** 10.1002/ctm2.1073

**Published:** 2022-10-20

**Authors:** Li Shao, Hong Zhao, Rongfang Guo, Jinlin Cheng, Xiaoyan Lu, Xiaohui Fan

**Affiliations:** ^1^ School of Clinical Medicine Hangzhou Normal University, The Affiliated Hospital of Hangzhou Normal University Hangzhou China; ^2^ Pharmaceutical Informatics Institute, College of Pharmaceutical Sciences Zhejiang University Hangzhou China; ^3^ The First Affiliated Hospital School of Medicine Zhejiang University Hangzhou China; ^4^ Innovation Center in Zhejiang University, State Key Laboratory of Component‐Based Chinese Medicine Hangzhou China; ^5^ Westlake Laboratory of Life Sciences and Biomedicine Hangzhou China

Dear Editor,

Chronic hepatitis B virus (HBV) infection affects about 844 million people worldwide, with two million annual deaths and a rising incidence. Persistent HBV infection may lead to liver inflammation and fibrosis, a leading risk for liver transplantation and hepatocellular carcinoma.^1^ However, few treatment options for controlling HBV‐related liver inflammation are currently available. The limited understanding on corresponding mechanisms impedes the discovery of rational therapeutics targeting on liver inflammation and fibrosis. Thus, we conducted a single‐cell transcriptomic study^2^ to better understand the mechanisms underlying chronic HBV‐related liver inflammation and to enable the discovery of therapeutic targets.

Liver biopsies from six chronic HBV patients with different grades of liver inflammation were included in the present study (Figure S1; Table S1). Single‐cell RNA sequencing of biopsied liver tissues was performed. Functions and cross‐talks of single cells were also investigated (provided in Supplementary Materials).

Based on around 24,000 single cells, we obtained 12 cell clusters. Among them, T cell population was the most abundant (Figure 1; Figure S2). By assigning the samples to different grades of inflammation (G), no apparent perturbation was observed in respect to the fractions of cells between grades G1 and G2 (Table S2). We further classified the 16, 386 T cells into nine cell subclusters, six of which varied significantly as liver inflammation progresses (Figure 2A,B). We then assigned them to known lineages based on marker genes (Figure 2C,D; Table S3). Concisely, T7 and T6 were annotated as CD8^+^ mucosal‐associated invariant T (MAIT) cells. T2 and T0 were identified as activated and exhausted CD8^+^ T cells, respectively. Most interestingly, MAIT cells, especially T7, decreased, while the ratio of T6 versus T7 increased in grade G2. Moreover, T7 dominated in grade G1 (95.20%) (Figure 2E and Figure S3A,B). The above findings were confirmed by additional single‐cell RNA sequencing data from liver samples of HBV‐infected patients with grades G1 (*n* = 4) and G2 (*n* = 4) (Figure S3C,D).^2^ T6 was assumed to be developed from T7 (Figure 2F), with genes such as *CCL3L1*, *PTMS*, *KLRG1* and *GADD45B* responsible for the transition (Figure S3E). Additionally, macrophage subcluster Ma3, inclined to have M2 signature, increased in grade G2 (Figure S4). Although no apparent perturbations were observed for the fractions of different cell types, T cell subpopulations were remodeled during the progression from grades G1 to G2.

**FIGURE 1:**
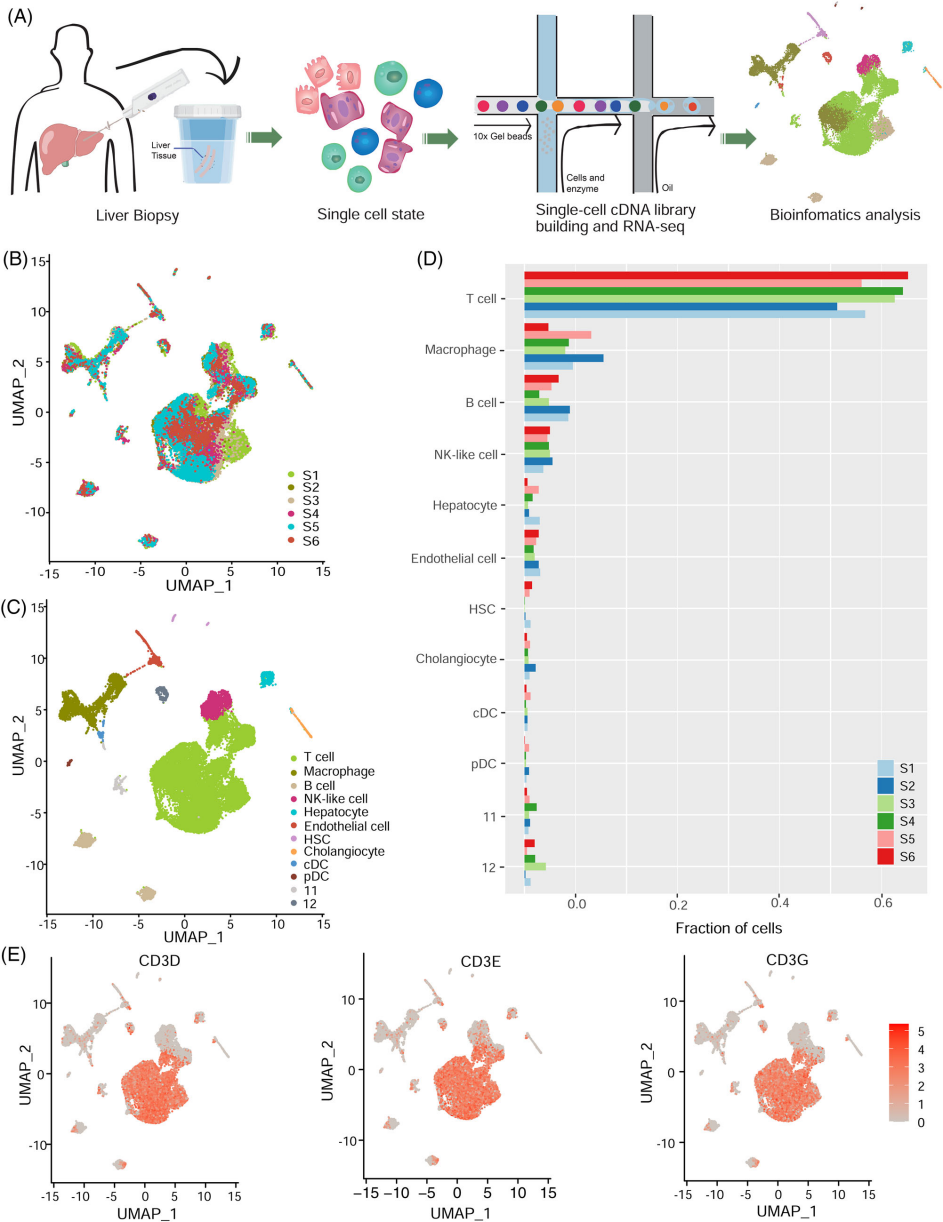
scRNA‐seq reveals cell types in liver biopsies of chronic hepatitis B virus (HBV) patients. (A) A schematic diagram of single‐cell sequencing for liver biopsies. (B) uniform manifold approximation and projection (UMAP) plots of single‐cell clusters coloured by samples. (C) UMAP plots of single‐cell clusters coloured by cell types. (D) Fraction of cells for all cell types in each sample. (E) Marker genes for T cells

**FIGURE 2:**
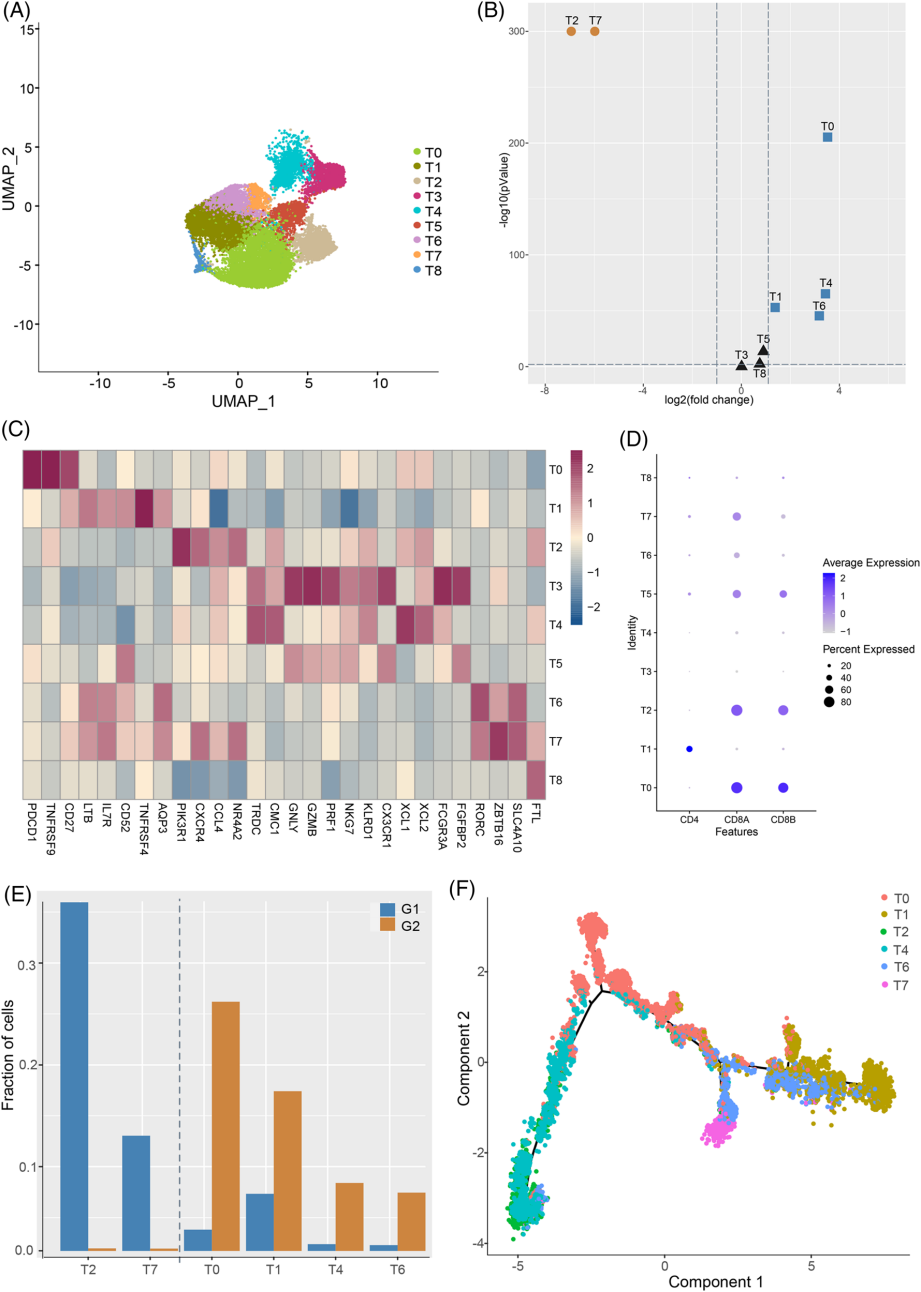
Cell populations fluctuate as inflammation progresses. (A) UMAP plots of T cell subclusters. (B) Volcano plot illustrating fold change and *p* values for T cell subclusters, where circles in grey mean insignificant. (C) Heatmap showing marker genes of T cell clusters. (D) Dotplot showing the expression level of *CD4*, *CD8A* and *CD8B* in different T cell subsets. (E) Fraction of cells for the six differential T cell subclusters coloured by grade. (F) Developmental trajectory for differential T cell subclusters

We further confirmed the presence of two MAIT cell populations by immunofluorescence, and annotated them as *CD3* ^+^ *SLC4A10* ^+^ *TNFAIP3* ^+^ (T7) and *CD3* ^+^ *SLC4A10* ^+^ *TNFAIP3* ^−^ (T6) MAIT cells (Figure 3). T7 may have stronger direct anti‐viral functions. Firstly, intrahepatic MAIT cells have been reported to be the most important innate effector cells secreting anti‐viral cytokine IFN‐γ,^3^ and T7 demonstrated up‐regulated pathways ‘interferon gamma secretion’ and ‘regulation of response to interferon gamma’ (Figure 4A). Secondly, T7 may have stronger type‐17 function of MAIT cell populations that might have a certain antiviral role,^4^ evidenced by the up‐regulation of pathways such as ‘T helper 17 type immune response’. Moreover, T7 may be stronger mediators of immune responses. On one hand, T7 may increase the anti‐viral capability of hepatocytes by ligand‐receptor pairs such as ‘FcRn complex_ALB’ (Figure 4B), where FcRn was reported to increase the sensitivity of hepatocyte to toxicity.^5^ Pathways such as ‘Toll‐like receptor 4 signaling pathways’ that can reduce HBV replication in an IFN‐independent manner in hepatocytes^6^ was also up‐regulated in T7. On the other hand, T7 may be more active in cytokine production and recruiting activated CD4^+^ and CD8^+^ T cells,^7^ evidenced by up‐regulated pathways such as ‘positive regulation of CD4 positive alpha beta T cell activation’. Then, T7 may assist virus infection control via activating NK cell and CD8^+^ T cell mediated immune responses through cross‐talks for T7‐NK cells and T7‐T2, evidenced by ligand‐receptor pairs such as ‘HLA‐C_KIR2DL3’ and ‘HLA‐A_KIR3DL1’, which were reported to function as helper masters to antiviral T cell responses.^8^ Such results indicated that MAIT cell population T7 may have active antiviral functions for reducing HBV replication in grade G1. Immunofluorescence results for additional two biopsies obtained from two G1 patients (Figure S5) further validated the presence of T7 in grade G1.

**FIGURE 3:**
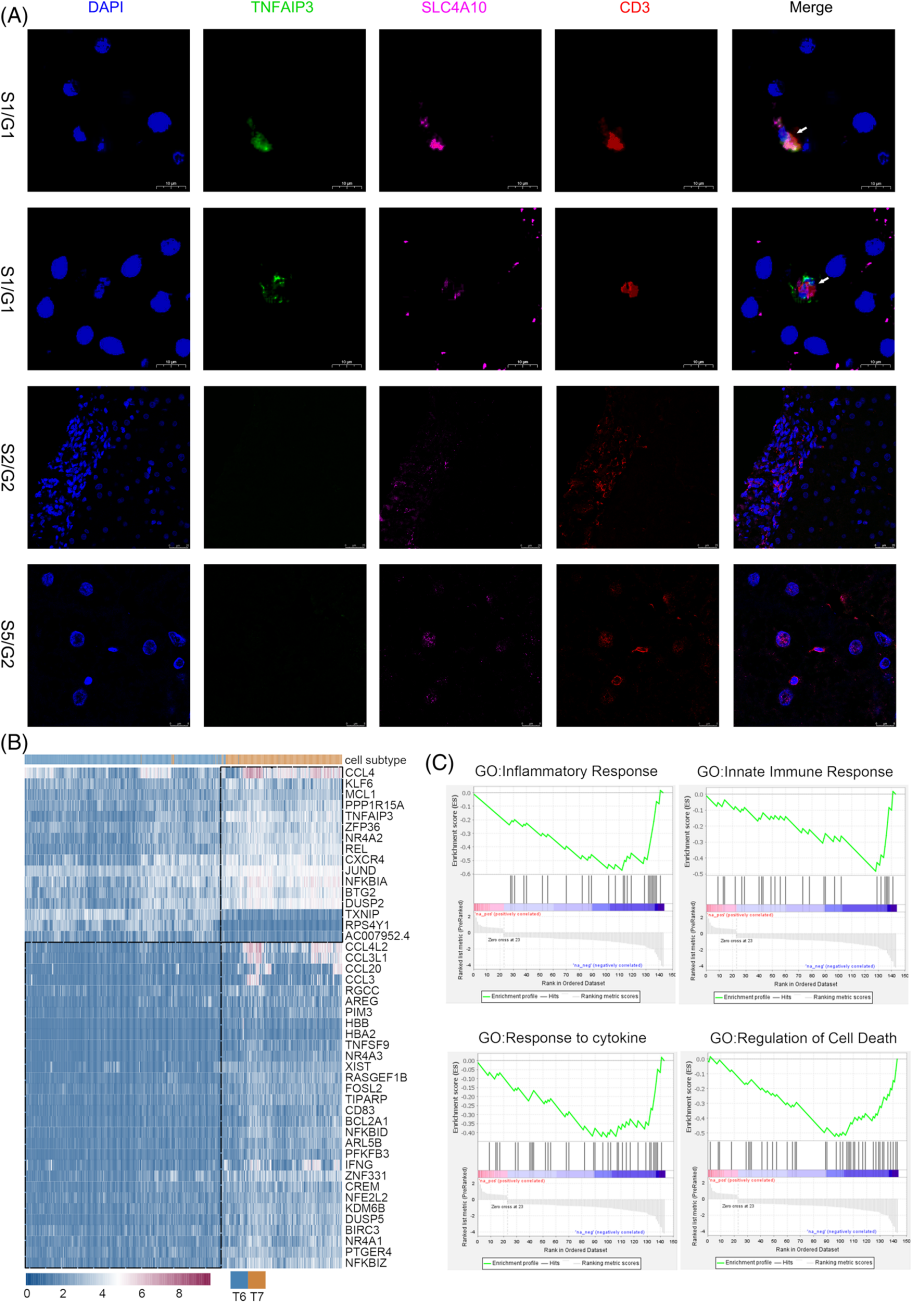
Characteristics of two mucosal‐associated invariant T (MAIT) cell populations. (A) Immunofluorescence of genes *CD3* (red), *SLC4A10* (rose red), *DAPI* (blue) and *TNFAIP3* (green) for liver biopsies obtained from samples S1 (grade G1), S2 (grade G2) and S5 (grade G2). (B) Heatmap of differential genes between two MAIT cell populations. (C) gene set enrichment analysis (GSEA) enriched terms for the genes differentially expressed between two MAIT cell populations

**FIGURE 4:**
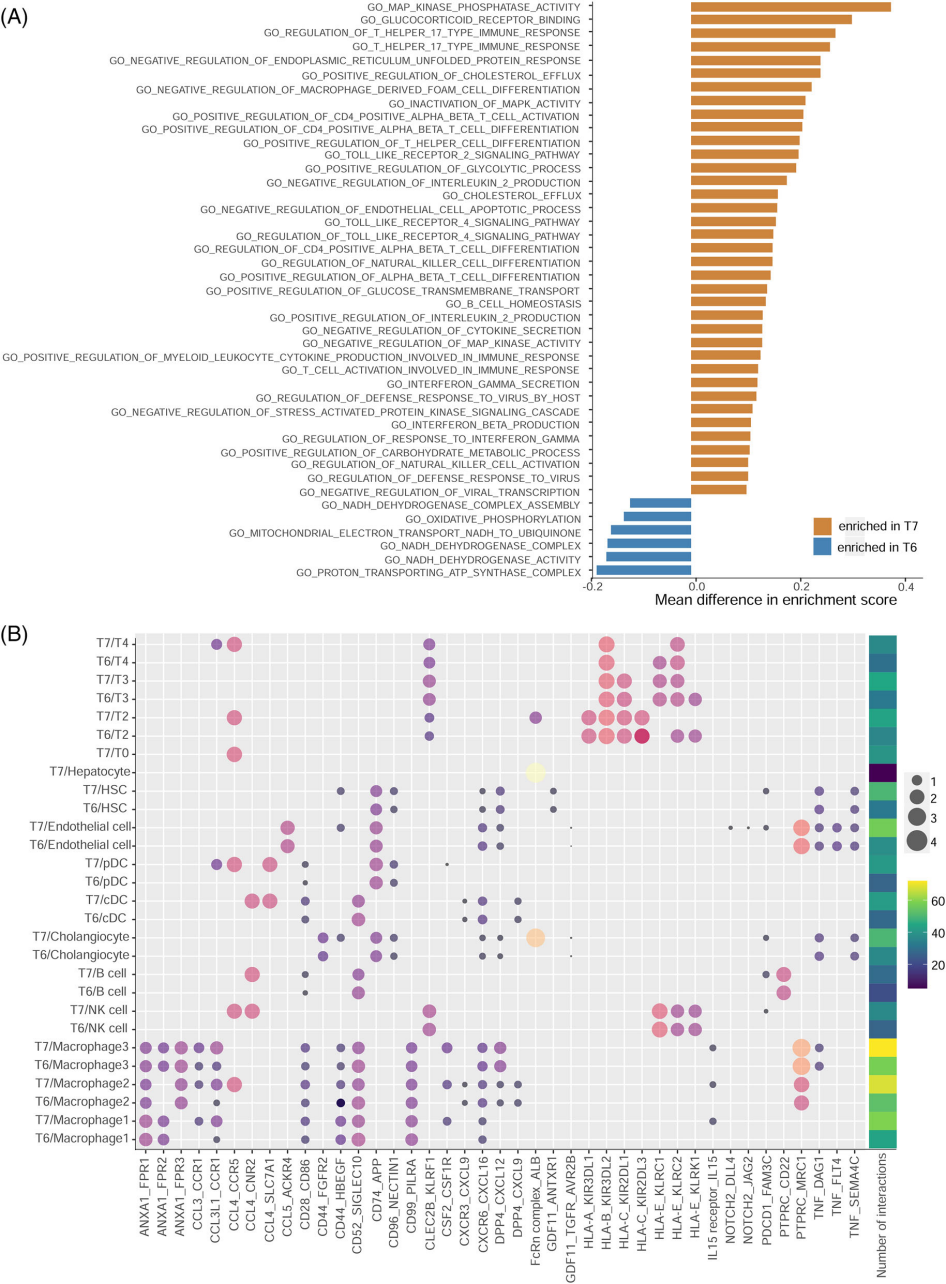
Gene set variation analysis (GSVA) and cross‐talk among T cell subclusters. (A) Variation of pathways for two mucosal‐associated invariant T (MAIT) cell populations using GSVA. (B) The number of interactions and ligand‐receptor pairs between MAIT cell subclusters (T7, T6) and others

We speculated impaired antiviral roles of T6 in grade G2 from the down‐regulation of corresponding pathways mentioned above, and the decrease in pathways ‘regulation of defense response to virus’ and ‘innate immune response’ (Figure 4A). Such results implied the existence of MAIT cell dysfunction in grade G2. We also observed a sharp decrease of activated CD8^+^ T cells, and a sharp increase of CD8^+^ exhausted T cells in grade G2. T cell exhaustion, in together with immune dysfunction of T6, may contribute to the inadequate HBV‐specific immune responses and inadequate infection control in grade G2. The increased M2‐type macrophage activation (Ma3) also implied the impaired immune response^9^ in grade G2. Bacterial superantigens resulting from the leaky gut of chronic HBV patients can make MAIT cells hyperactive and exhausted, which will lead to immunosuppression and increases the risk of secondary opportunistic infections.^10^ Thus, the dysfunction of MAIT cells T6 and exhaustion of CD8^+^ T cells may also provide an explanation for the progression of HBV‐related inflammation in grade G2. Moreover, MAIT cells demonstrated extensive interactions with other immune cells (provided in Supplementary Materials).

In conclusion, our study reported the remodelling of T cell subpopulations and the presence of two MAIT cell populations that may contribute to HBV‐related liver inflammation for the first time. MAIT cell population T7 may have active roles in assisting virus clearance via direct anti‐viral functions and mediating immune responses, which was impaired in T6, the proportion of which in *CD8* ^+^ MAIT cells increased in grade G2. The sharp decrease of active *CD8* ^+^ T cells, MAIT cell dysfunction in together may contribute to the inadequate HBV‐specific immune responses in grade G2. Inspired from the decrease of MAIT cells and increased ratio of T6 versus T7 during liver inflammation progression, MAIT cells may act as a potential target for controlling HBV‐related liver inflammation in future studies. However, studies using more samples are required to confirm the findings between G1 and G2 reported in this study.

## CONFLICT OF INTEREST

The authors declare that they have no conflict of interest.

## Supporting information


**Figure S1**. The H&E results for three representative liver biopsy samples.

**Figure S2**. Violin plots of maker genes for macrophage, B cell, NK‐like cell, hepatocyte, endothelial cell, HSC, cholangiocyte, cDC, pDC and T cell.

**Figure S3**. (A) The proportion of MAIT cells in CD8^+^ T cells. (B) The ratio of cell number proportions between T6 and T7 in CD8^+^ mucosal‐associated invariant T (MAIT) cells. (C) The proportion of MAIT cells in CD8^+^ T cells for validation dataset. (D) The ratio of cell number proportions between T6 and T7 in CD8^+^ MAIT cells for validation dataset. (E) The beam‐map illustrating genes responsible for the transition between T6 and T7.

**Figure S4**. (A) UMAP plot of three macrophage subclusters. (B) The fraction of cells in grades G1 and G2 for three macrophage subclusters. (C) M1 and M2 signature scores for each macrophage subcluster.

**Figure S5**. Immunofluorescence results for additional two biopsies obtained from two G1 patients

**Table S1**. Clinical and biochemical parameters for chronic hepatitis B virus (HBV) patients

**Table S2**. Fraction of cells in each cell type for six samples.

**Table S3**. Overview of T cell clusters and annotation

Supporting information1

Supporting information1
