# Supplementary figures and images for: Biopsy‐based single‐cell transcriptomics reveals MAIT cells as potential targets for controlling fibrosis‐related liver inflammation due to chronic hepatitis‐B infection
Source: Clin Transl Med. 2022 Oct 20;12(10):e1073. doi: 10.1002/ctm2.1073 (PMC9582669; doi:10.1002/ctm2.1073)

10X

20X

S1/G1

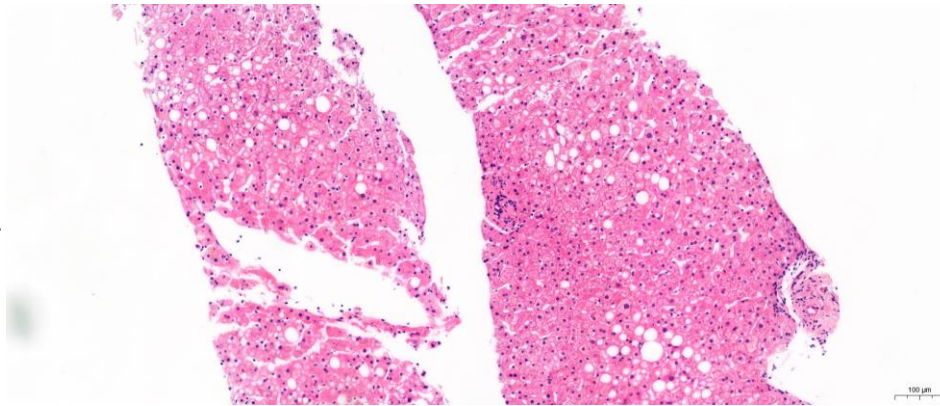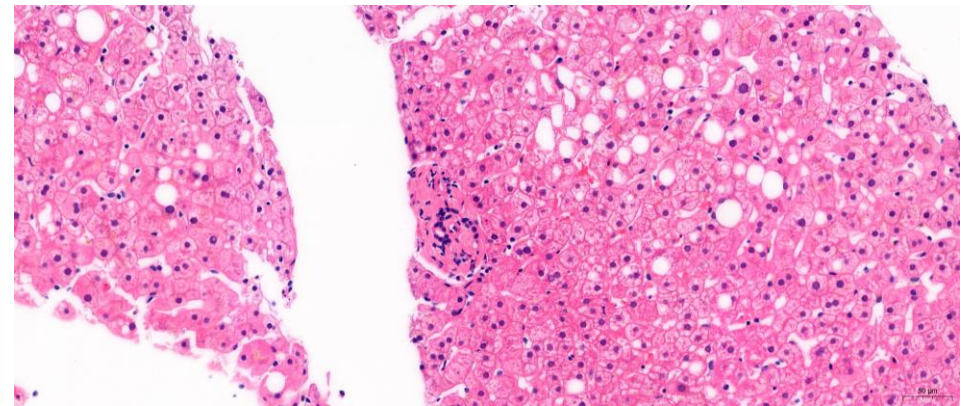

S2/G2

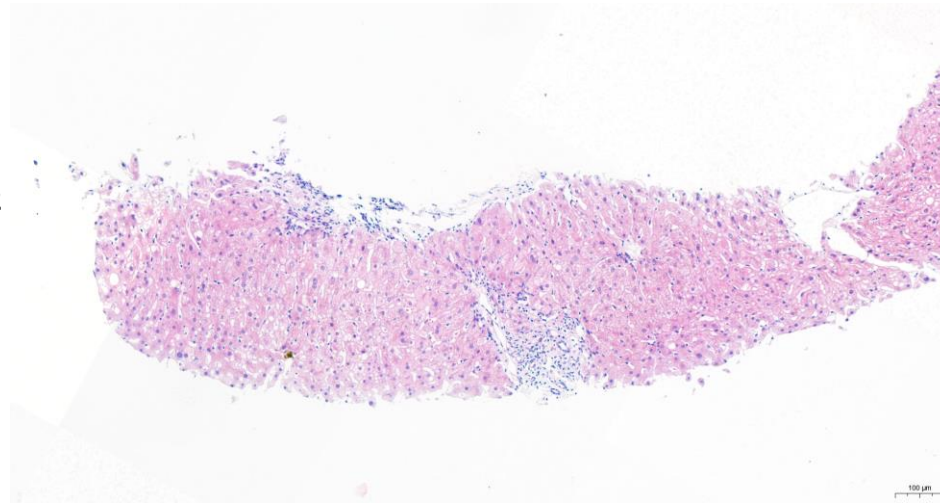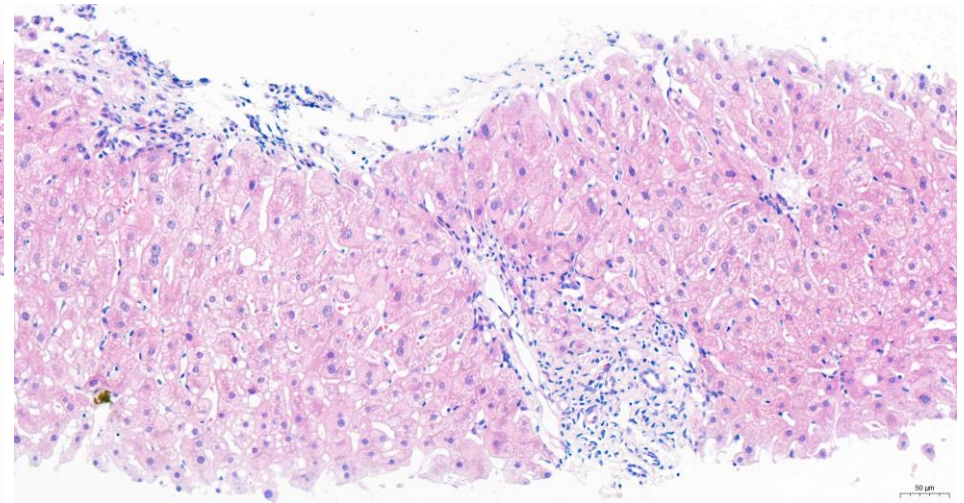

S5/G2

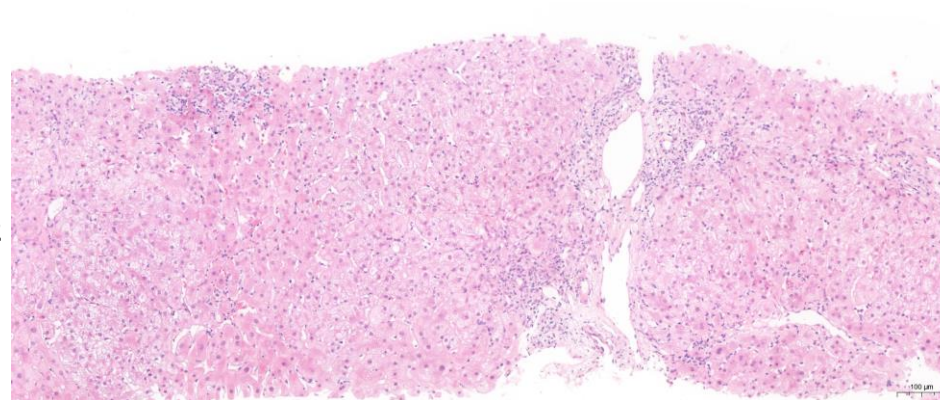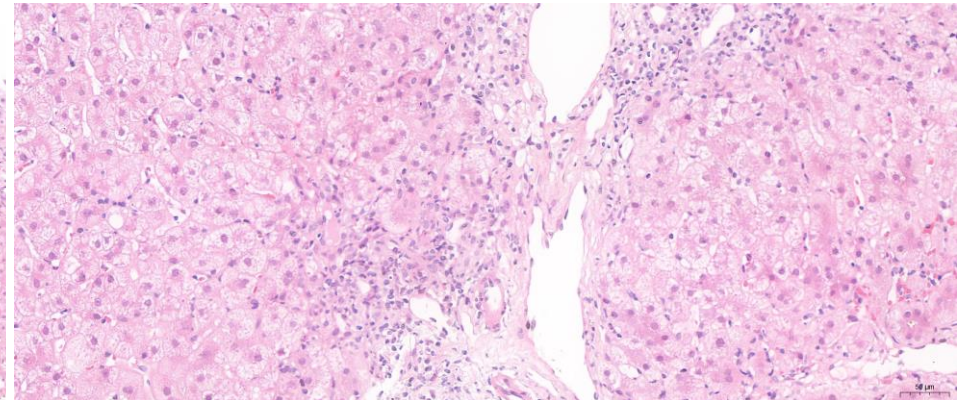

Supplement: Supplementary file 1 — Figure S1. The H&E results for three representative liver biopsy samples. [file CTM2-12-e1073-s006.pdf]

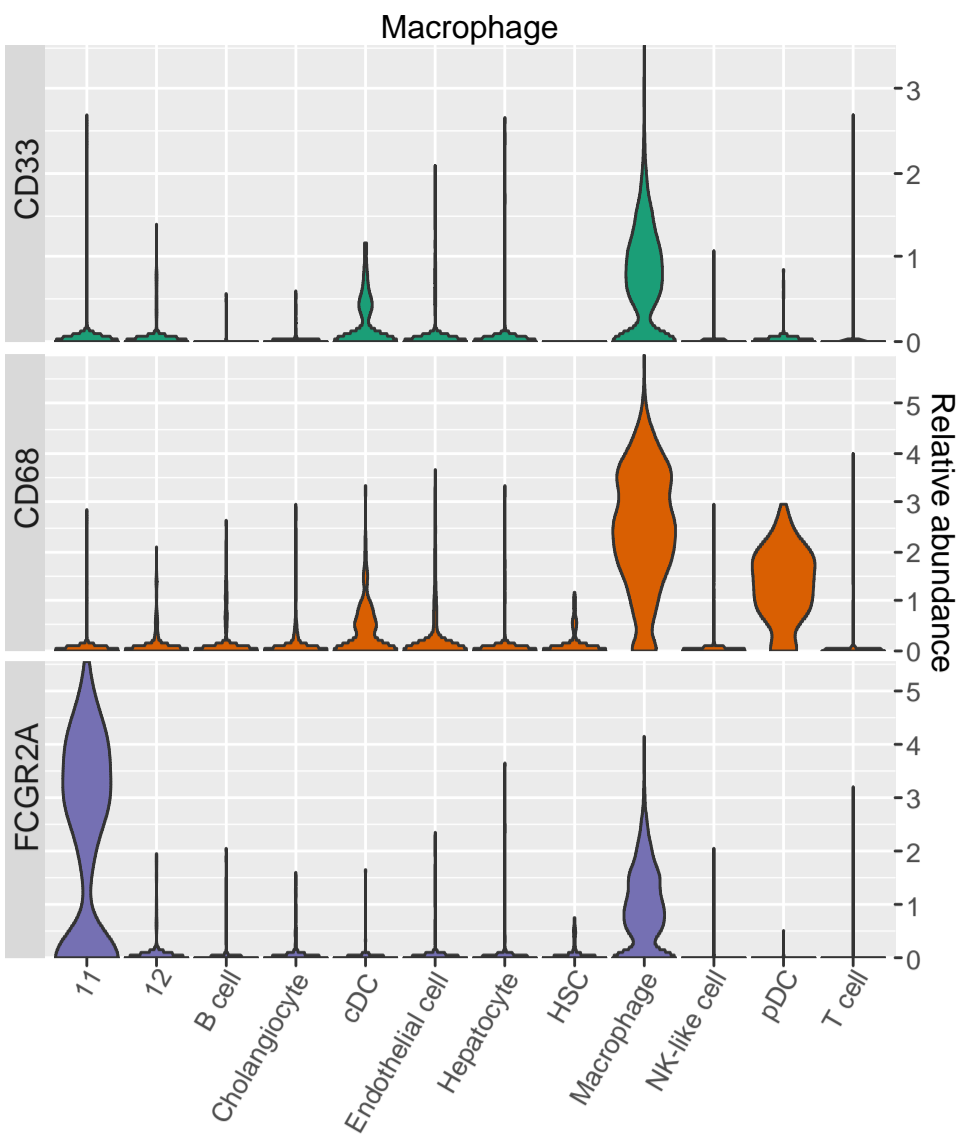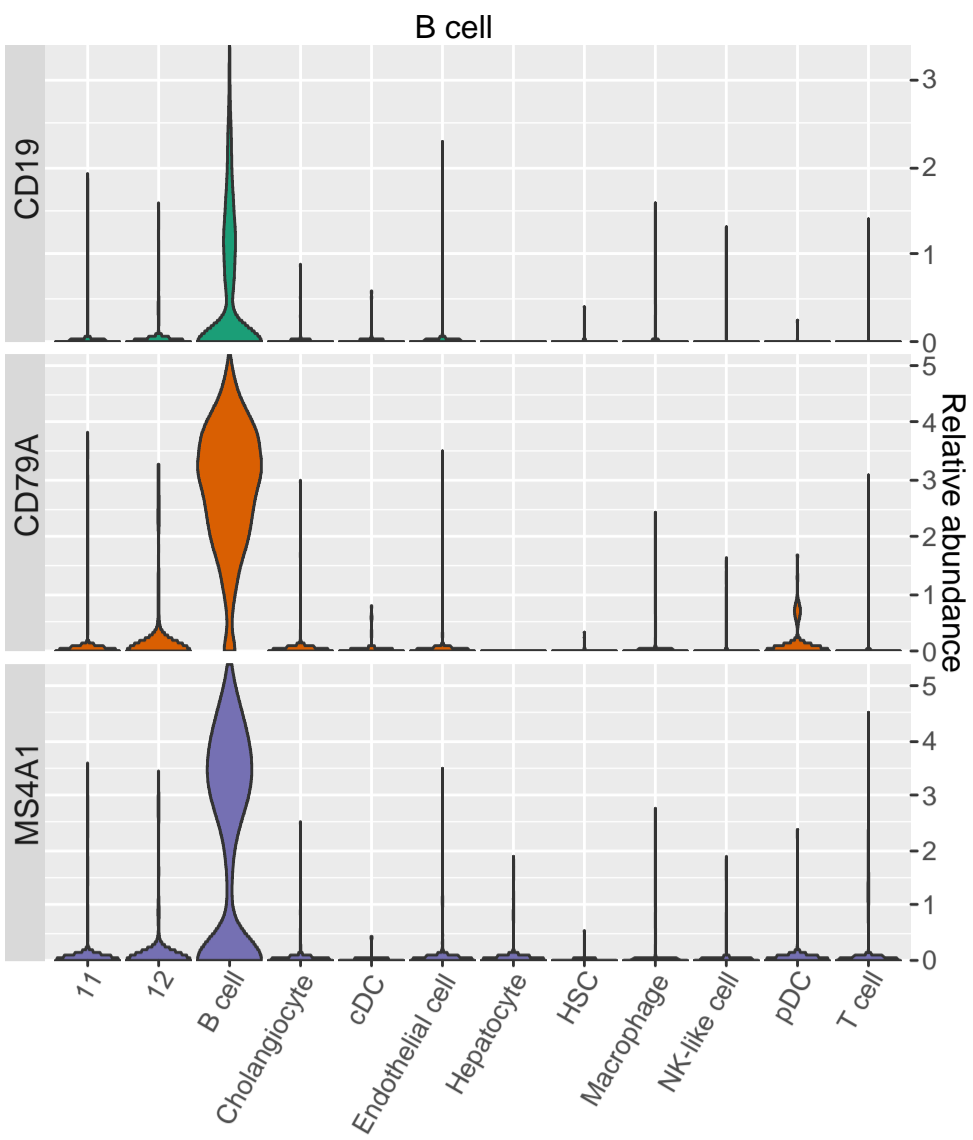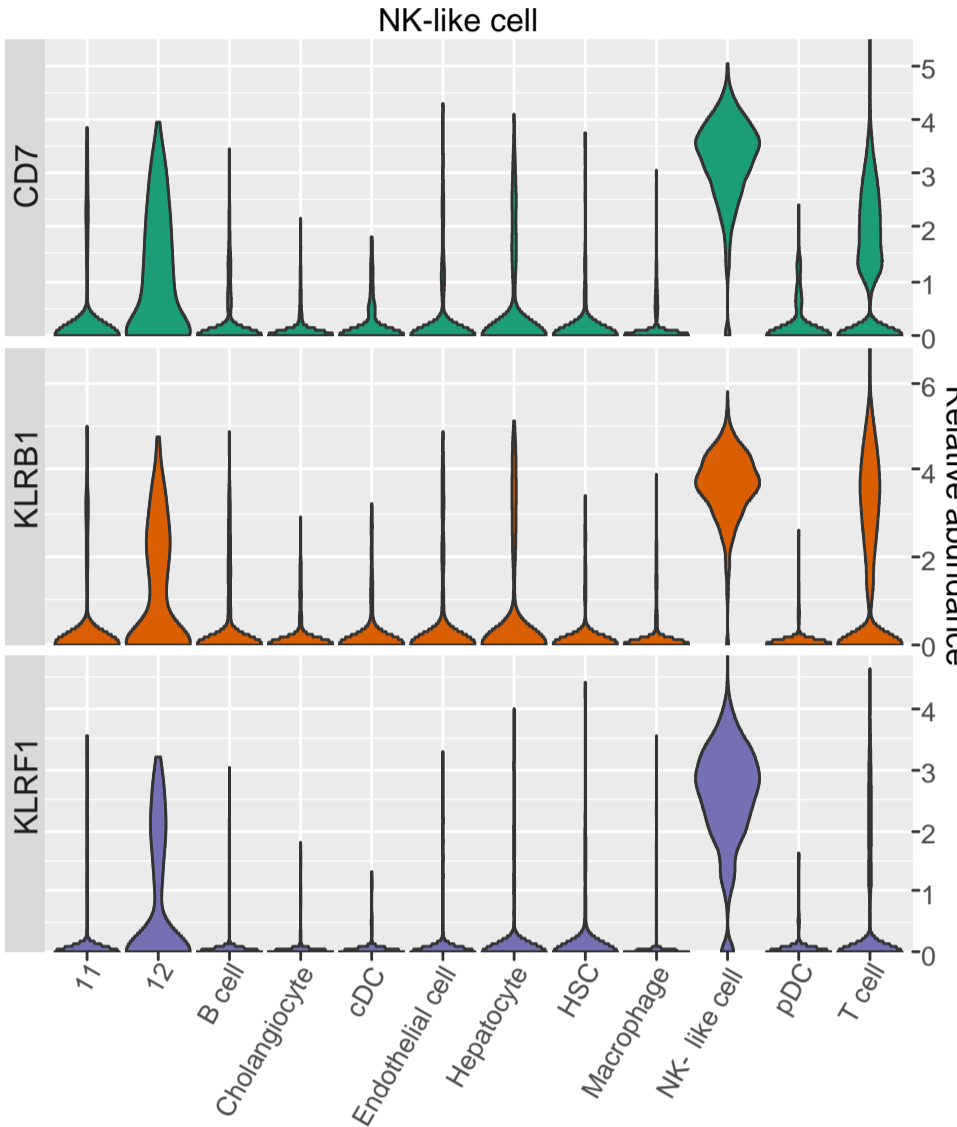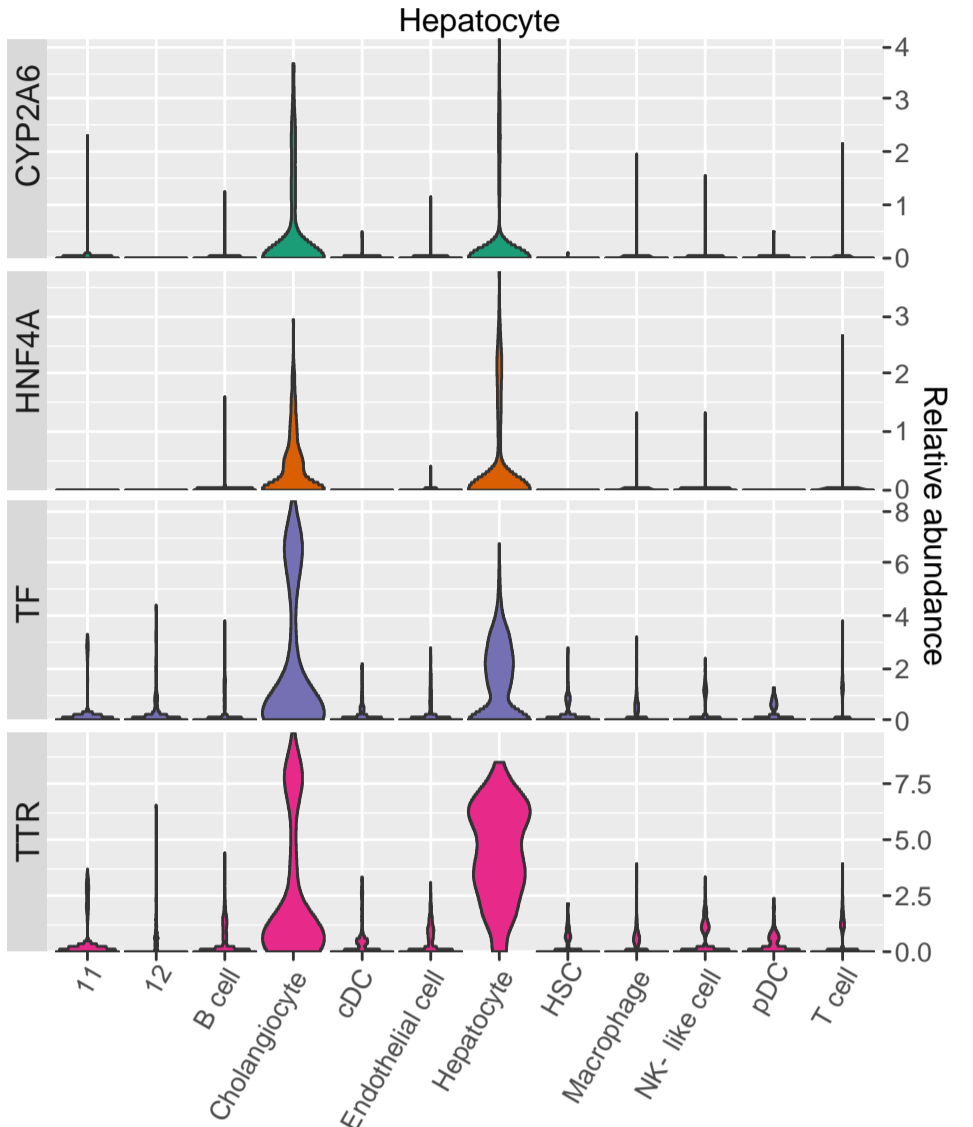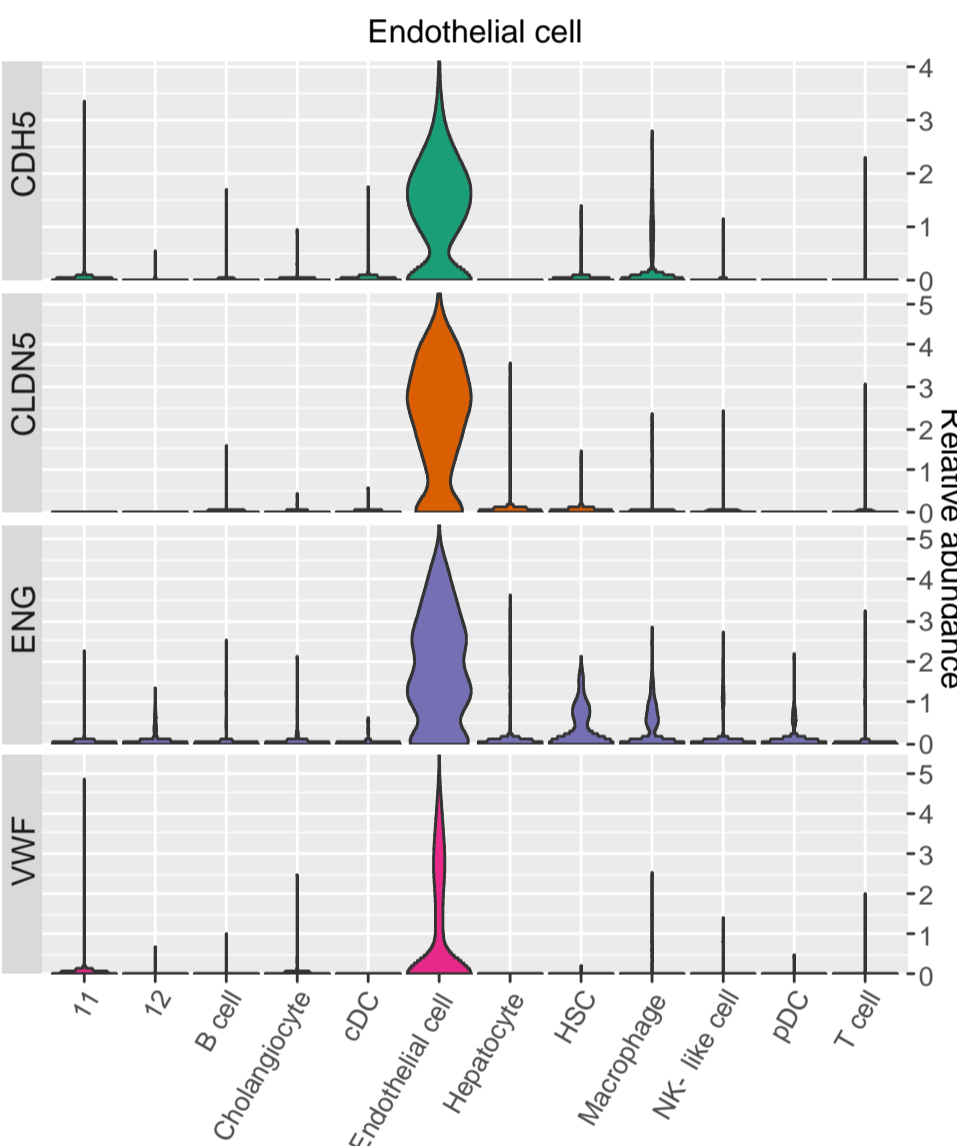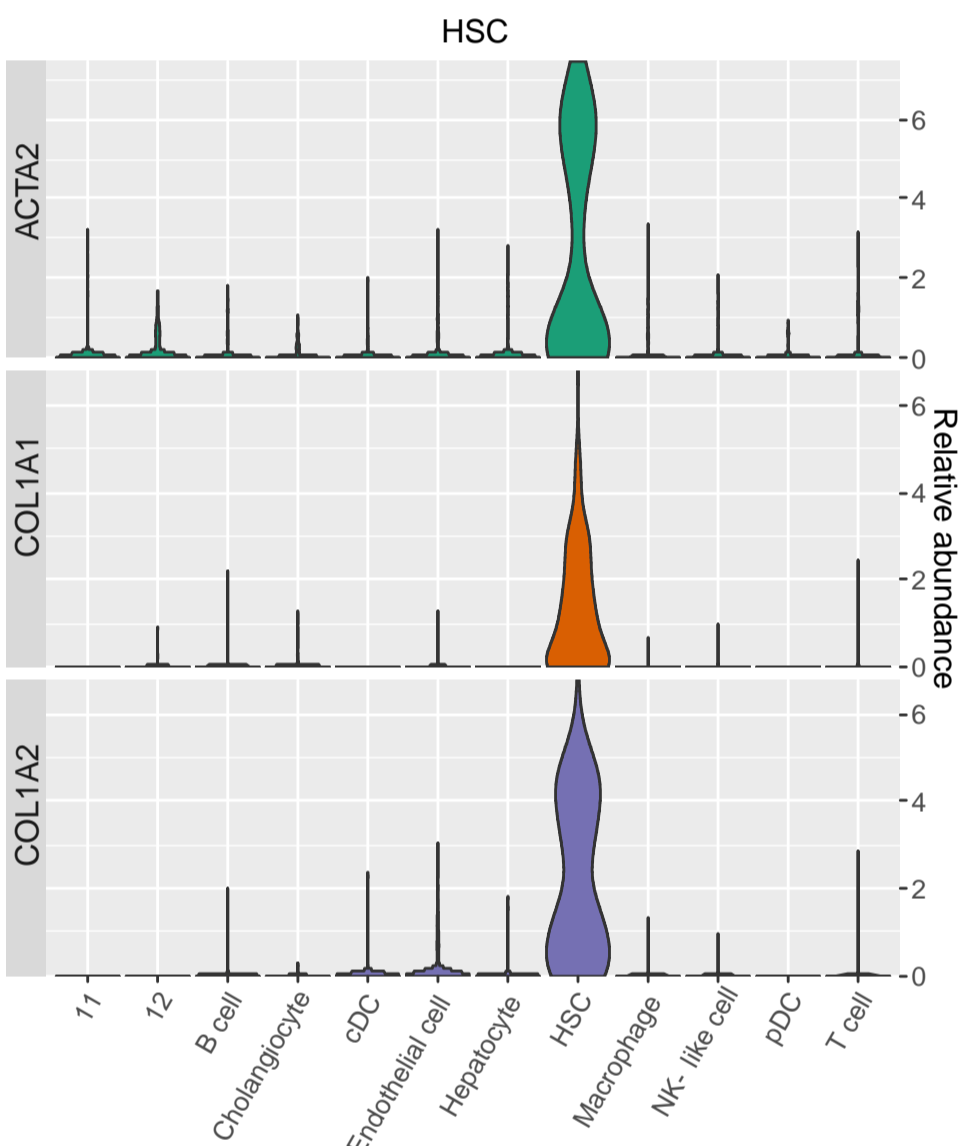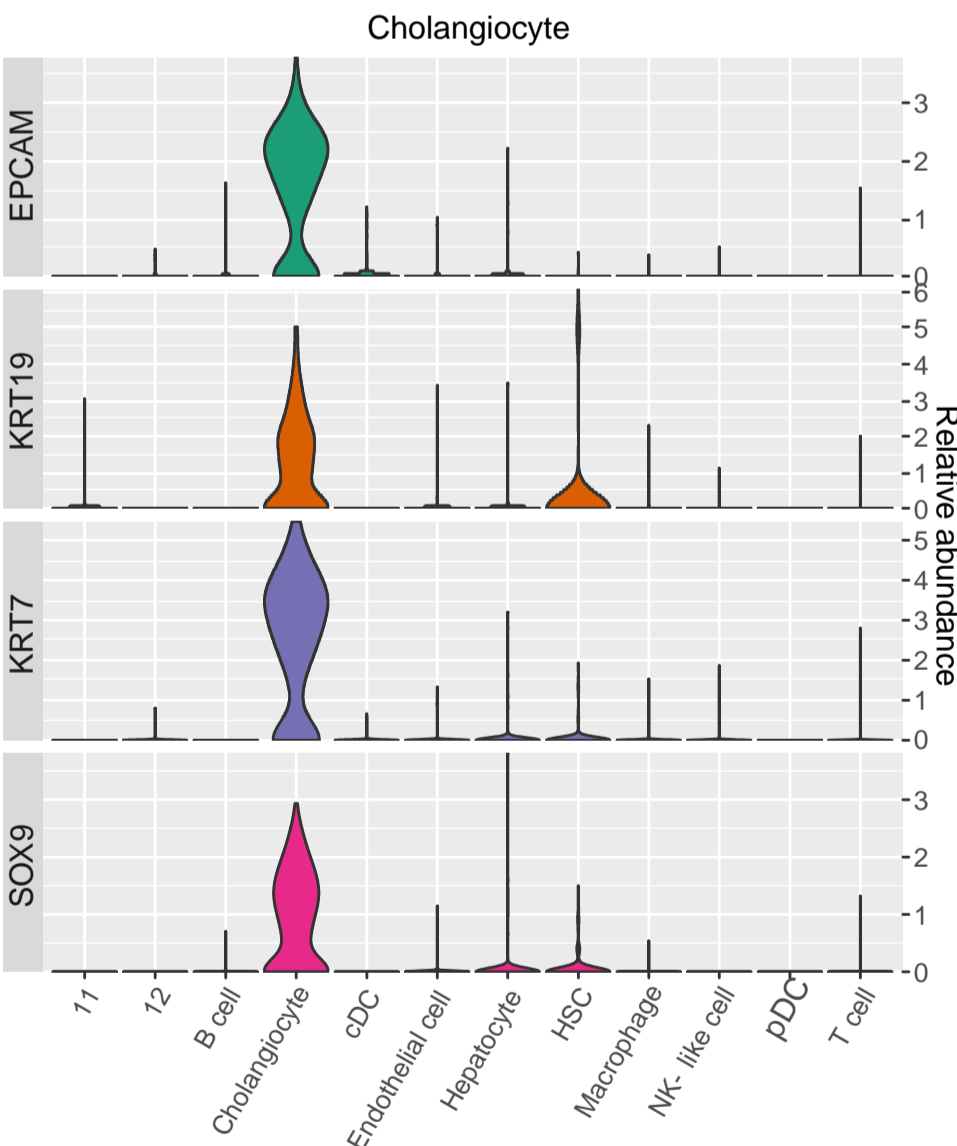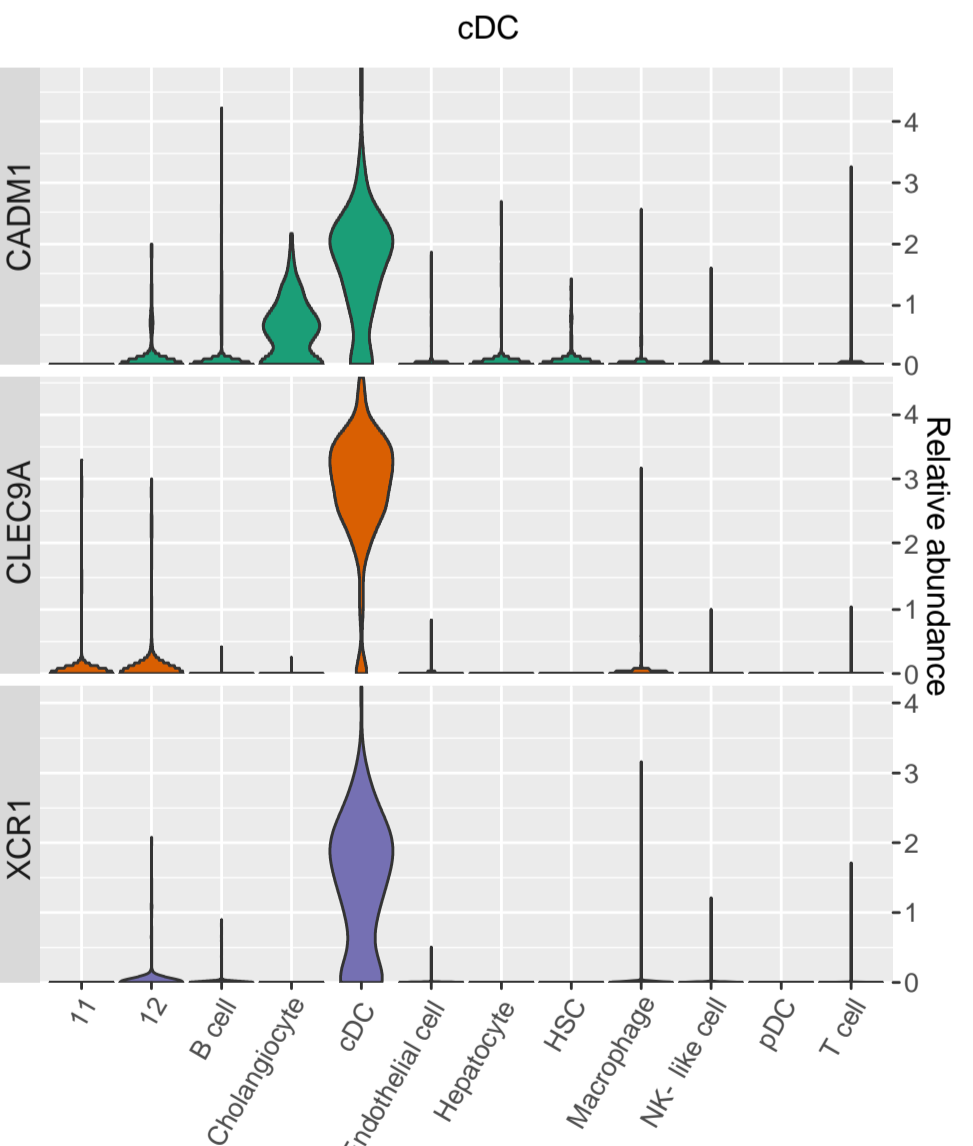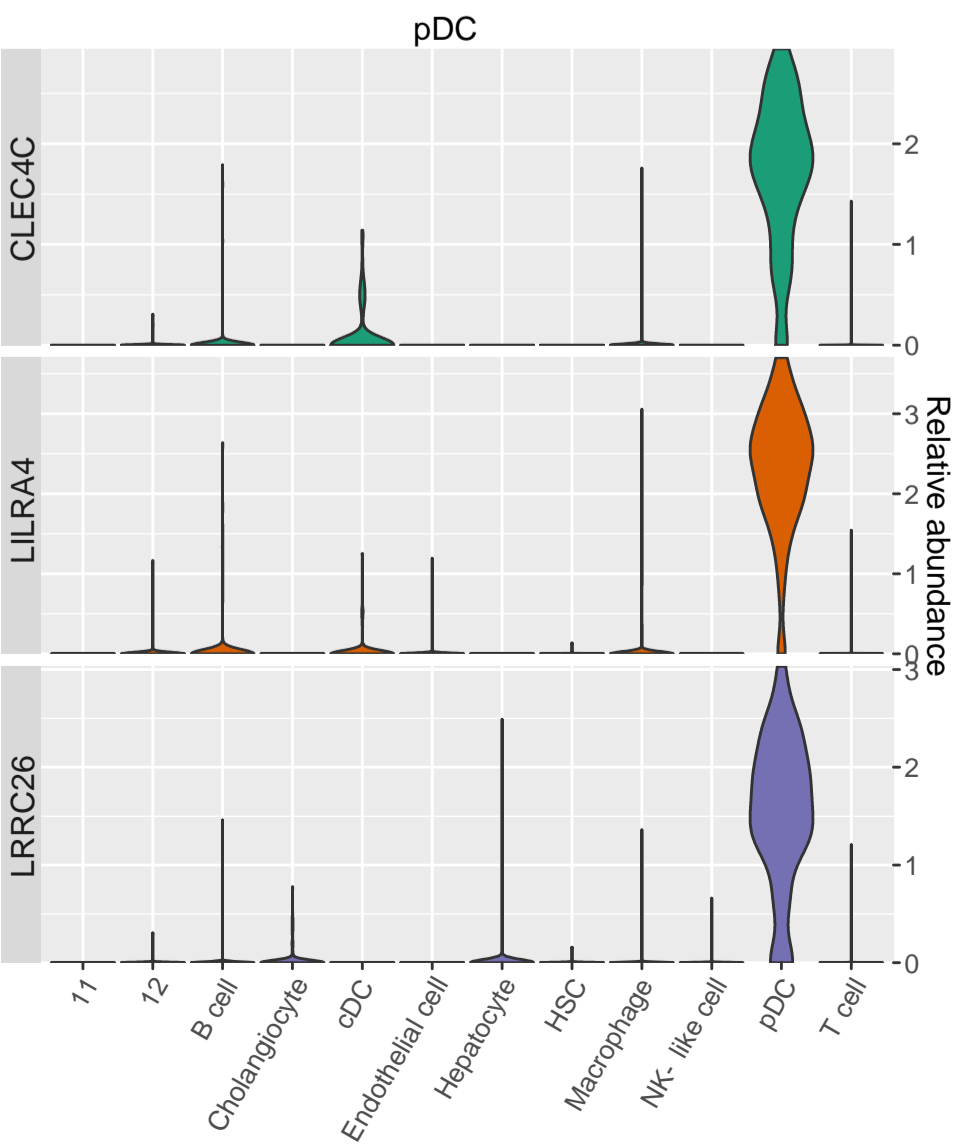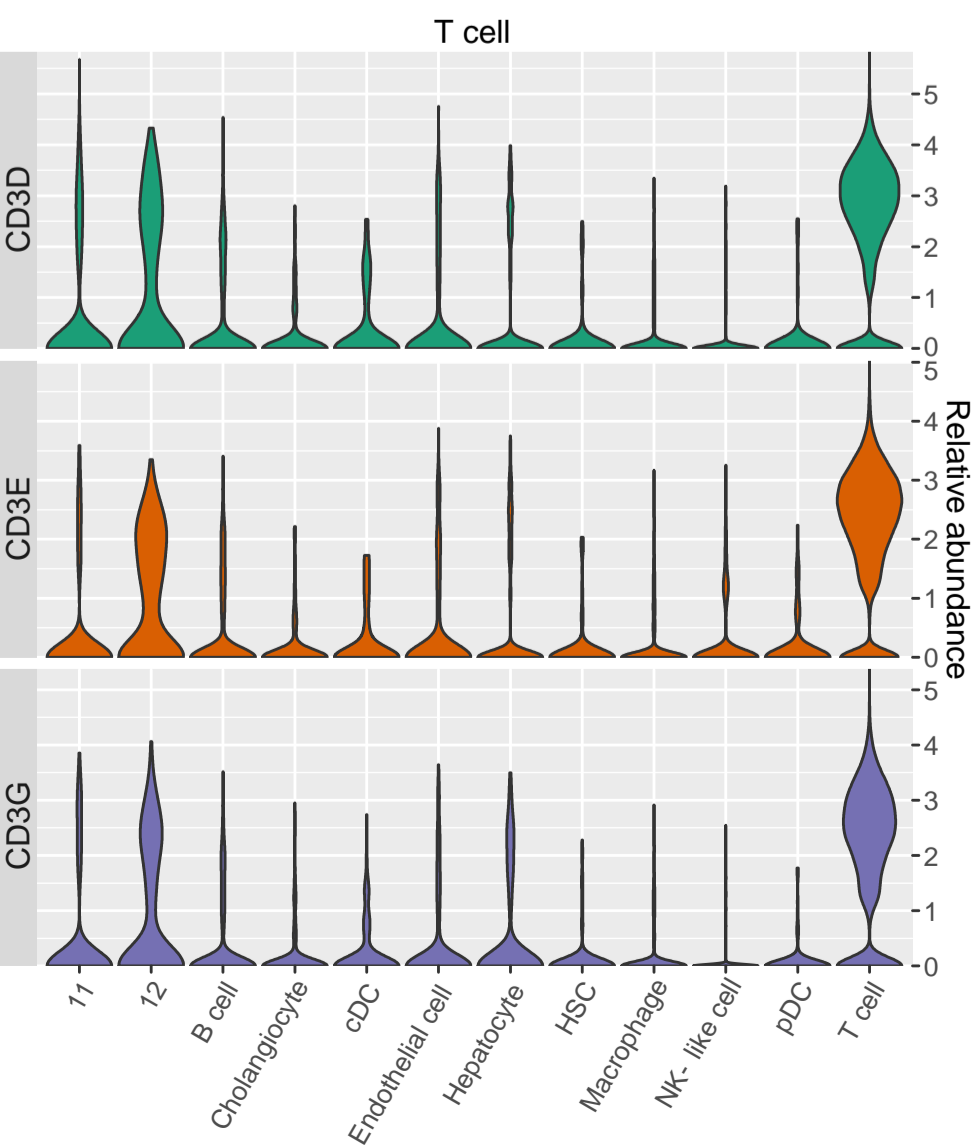

Supplement: Supplementary file 2 — Figure S2. Violin plots of maker genes for macrophage, B cell, NK‐like cell, hepatocyte, endothelial cell, HSC, cholangiocyte, cDC, pDC and T cell. [file CTM2-12-e1073-s010.pdf]

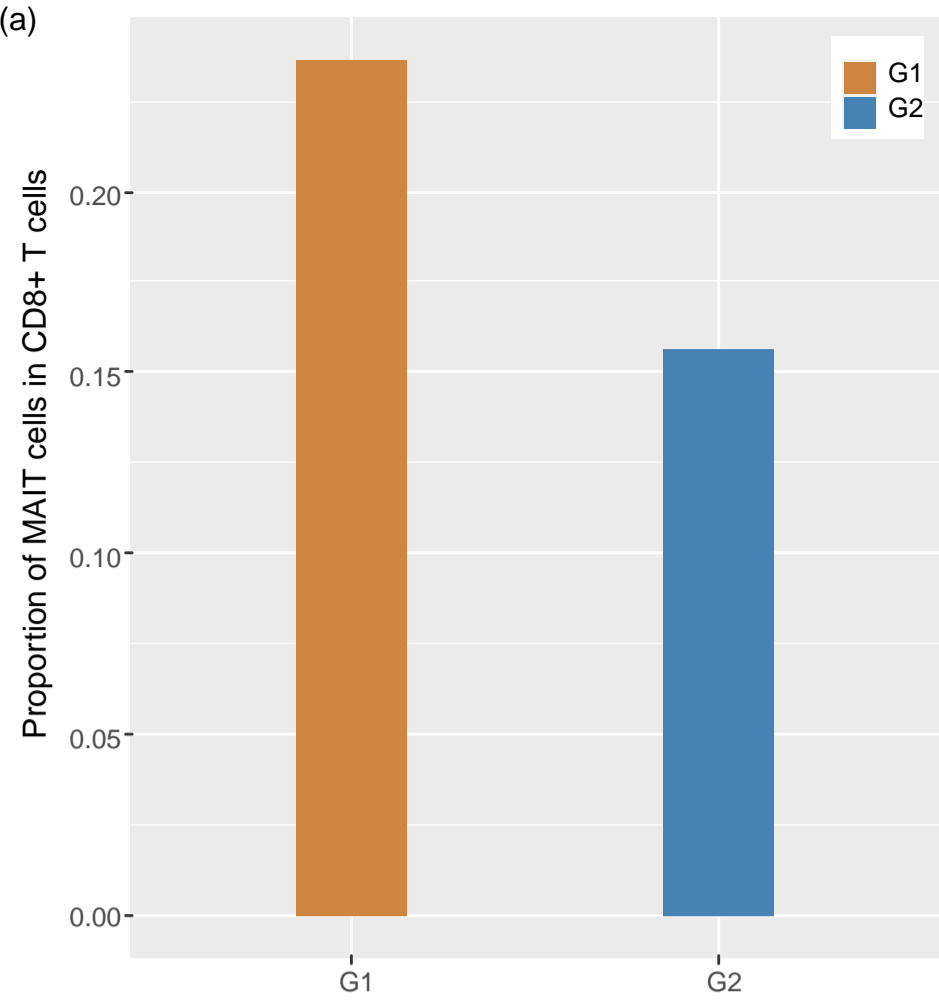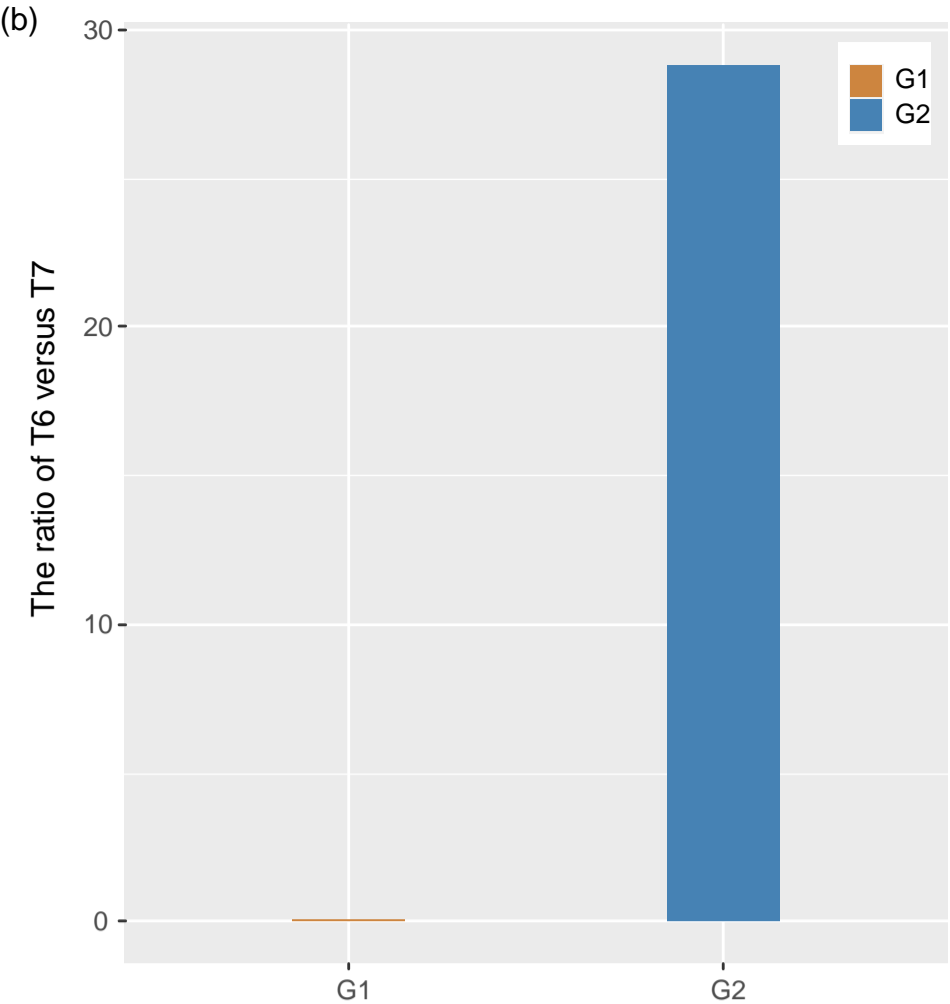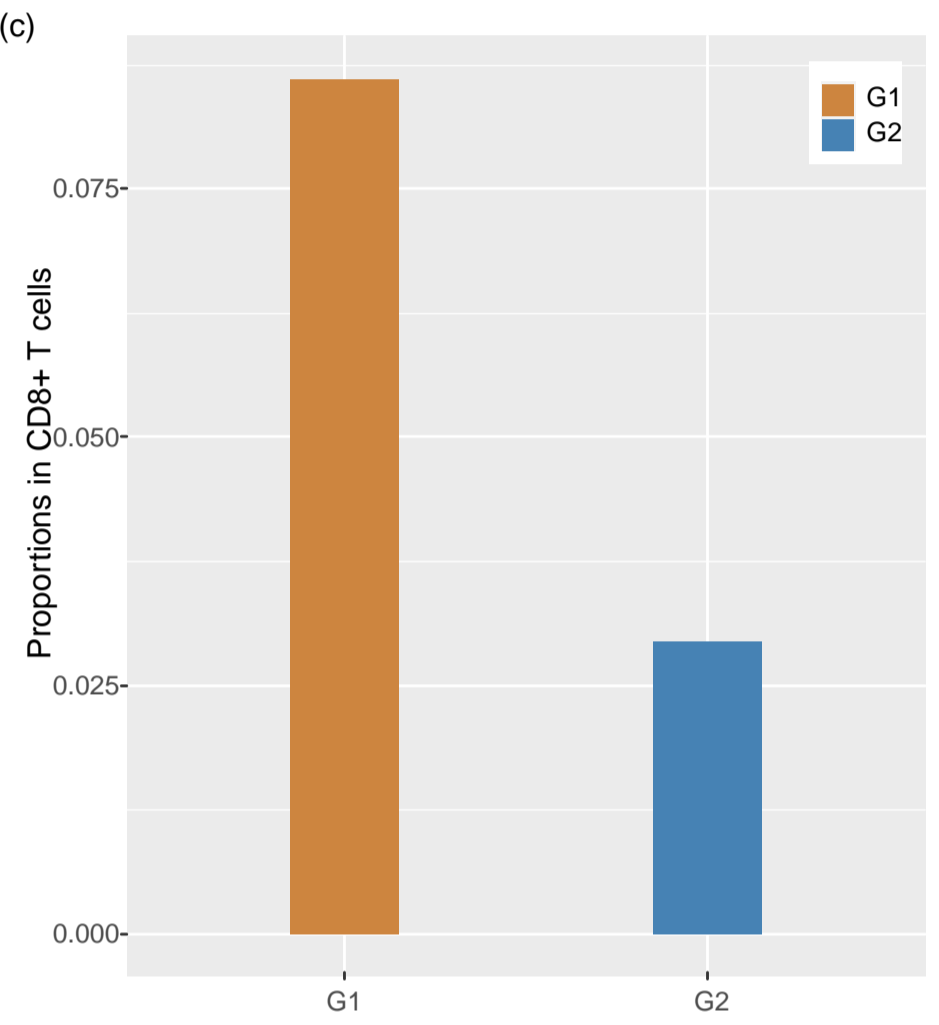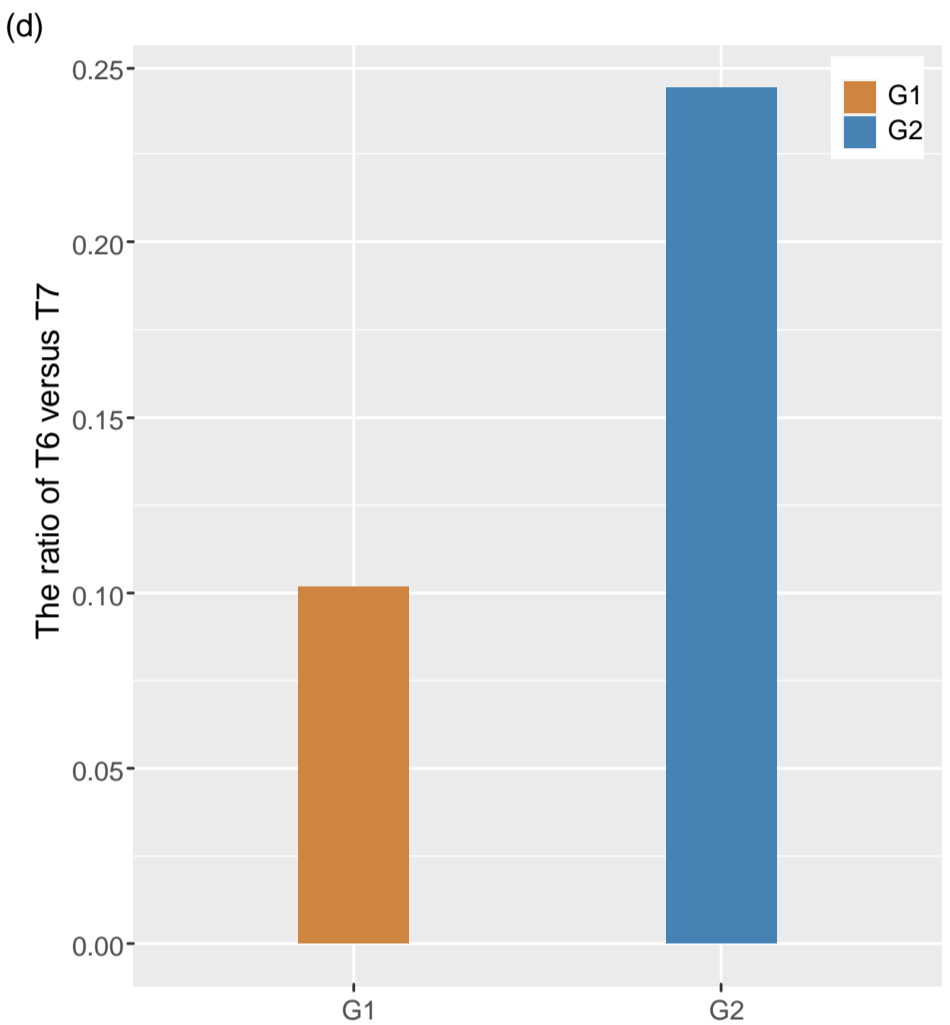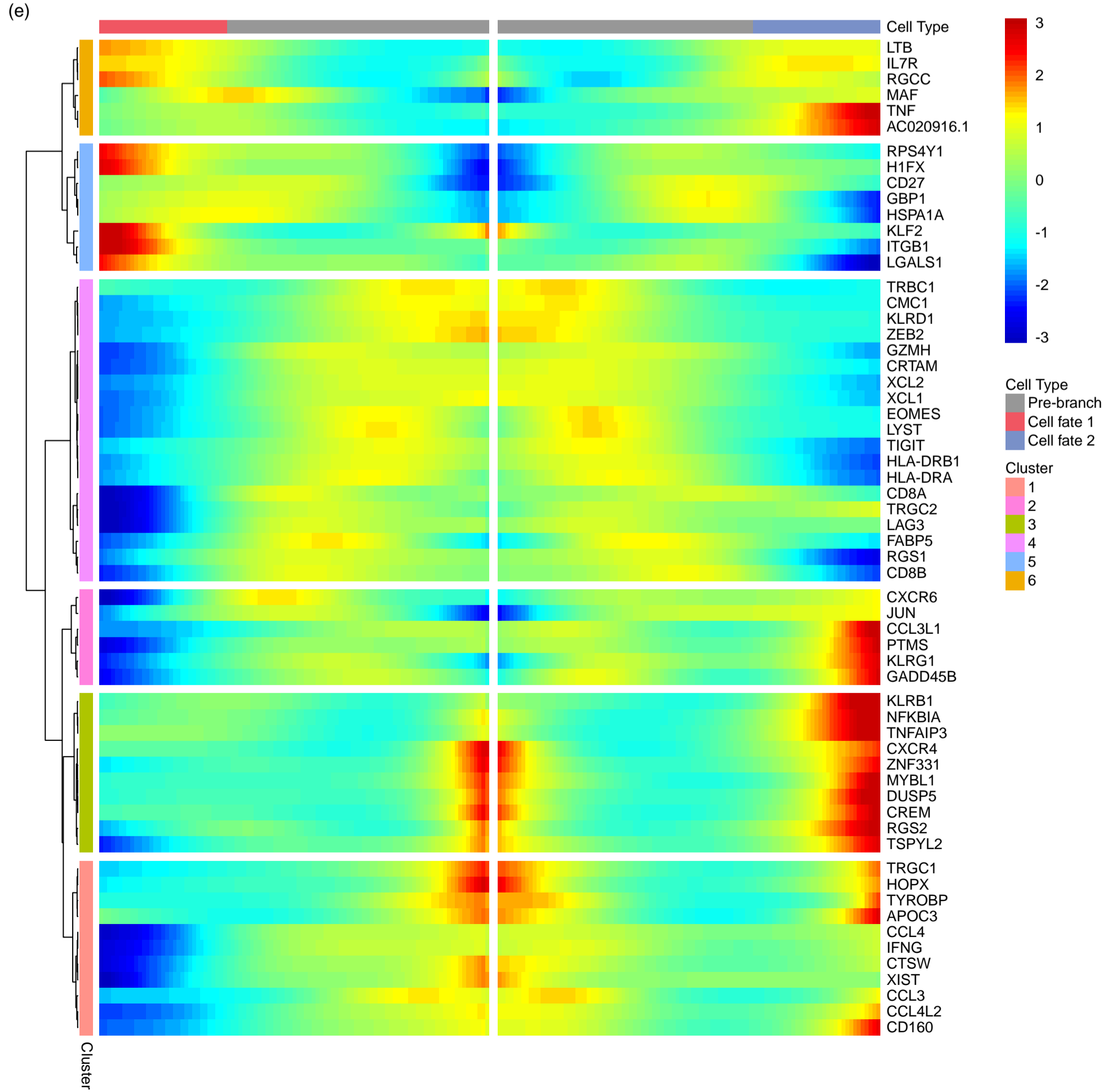

Supplement: Supplementary file 3 — Figure S3. (A) The proportion of MAIT cells in CD8+ T cells. (B) The ratio of cell number proportions between T6 and T7 in CD8+ mucosal‐associated invariant T (MAIT) cells. (C) The proportion of MAIT cells in CD8+ T cells for validation dataset. (D) The ratio of cell number proportions between T6 and T7 in CD8+ MAIT cells for validation dataset. (E) The beam‐map illustrating genes responsible for the transition between T6 and T7. [file CTM2-12-e1073-s003.pdf]

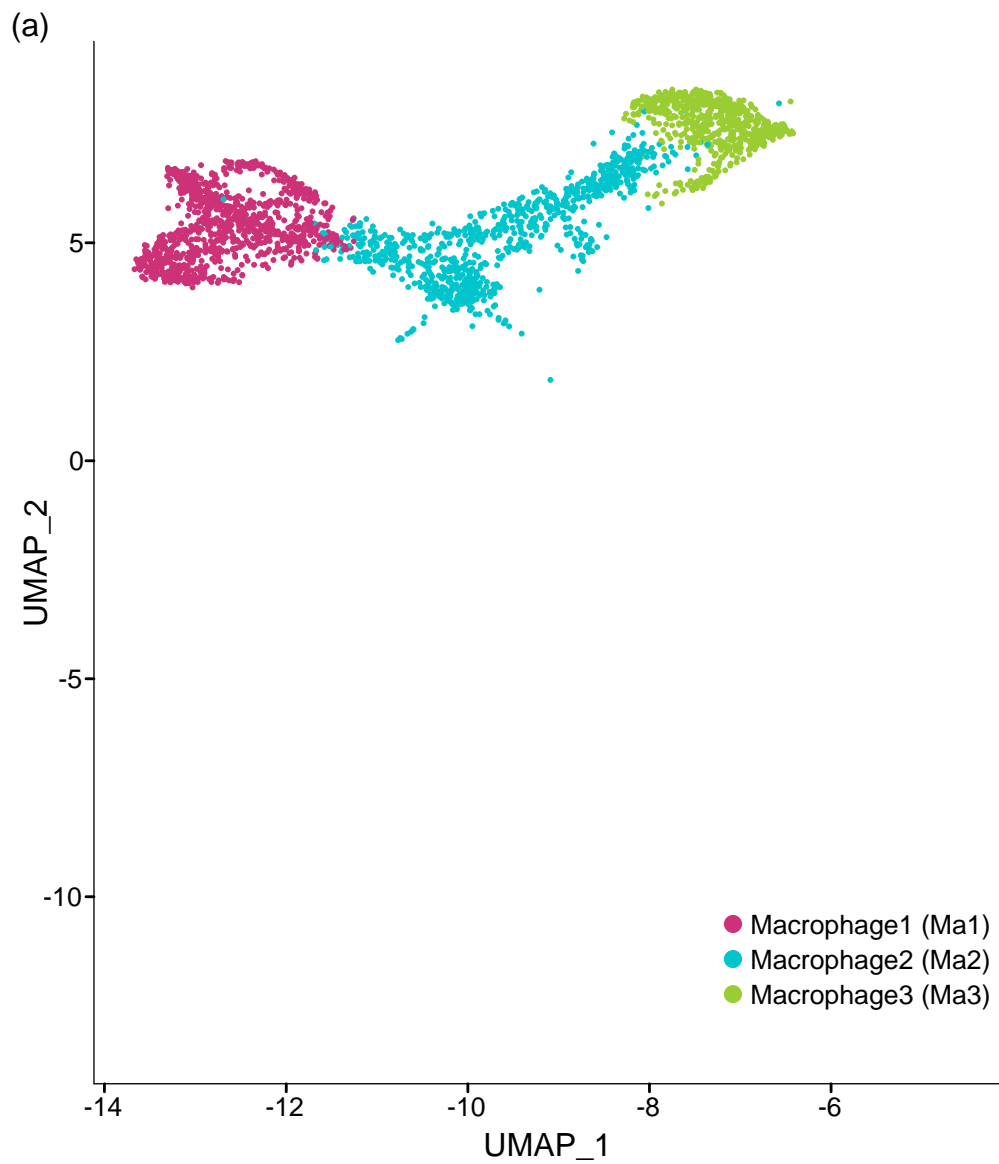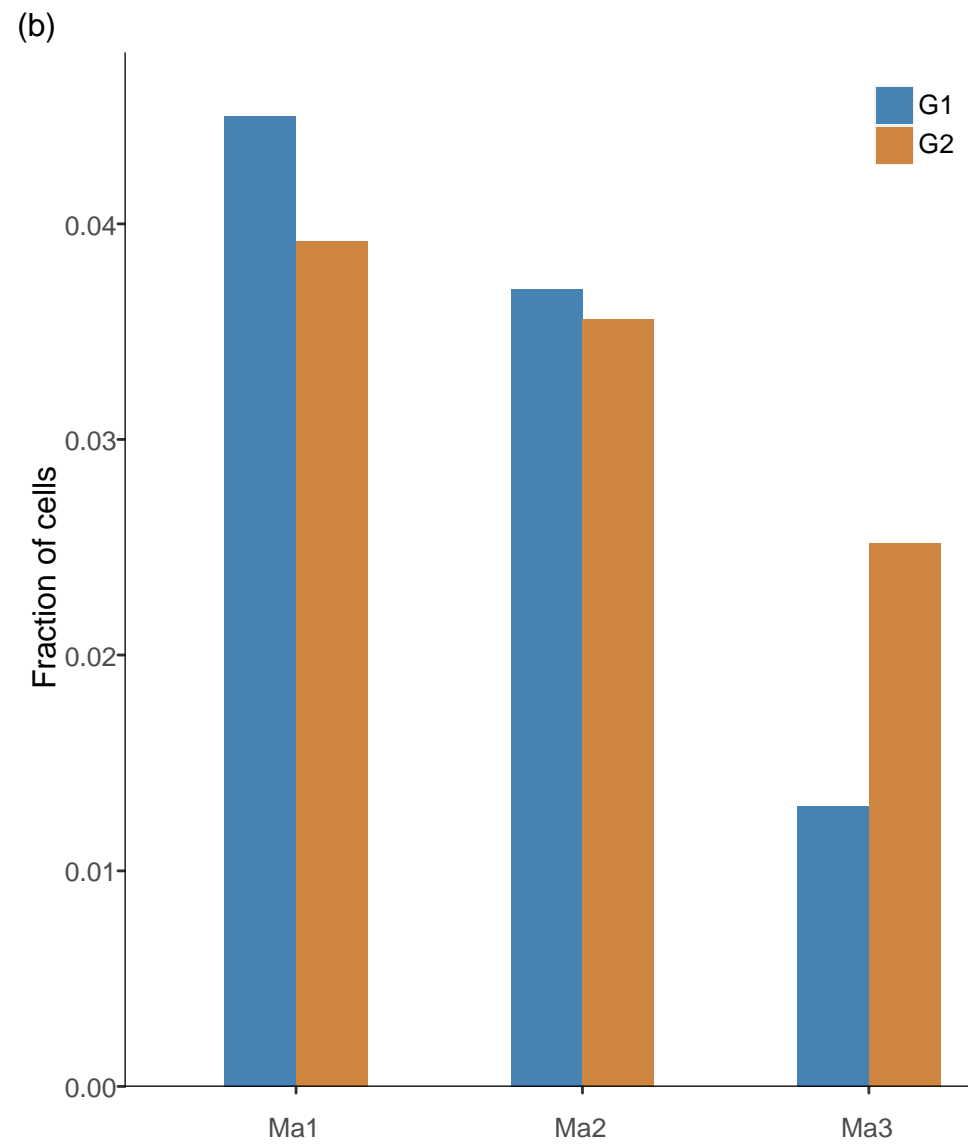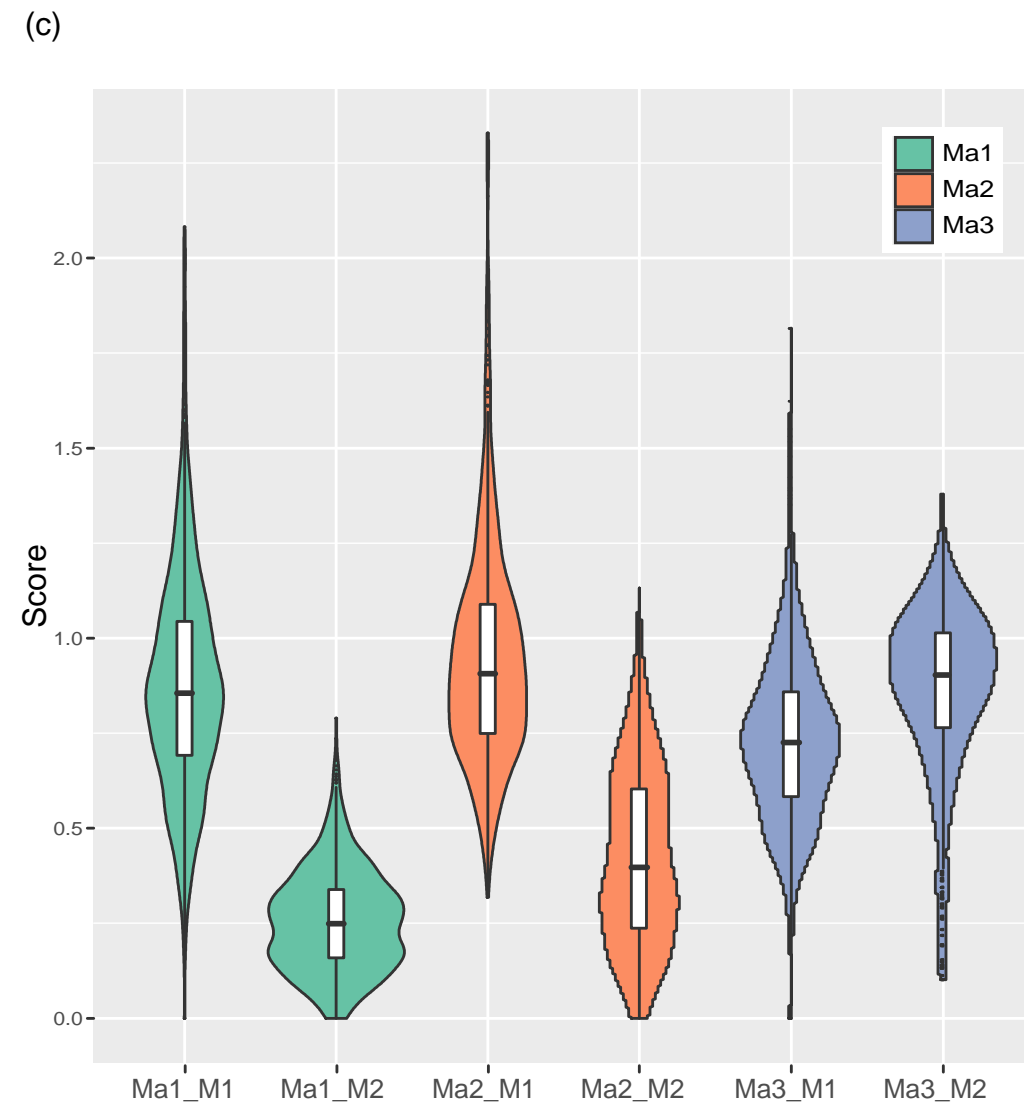

Supplement: Supplementary file 4 — Figure S4. (A) UMAP plot of three macrophage subclusters. (B) The fraction of cells in grades G1 and G2 for three macrophage subclusters. (C) M1 and M2 signature scores for each macrophage subcluster. [file CTM2-12-e1073-s001.pdf]

DAPI

TNFAIP3

SLC4A10

CD3

Merge

Additional S1/G1

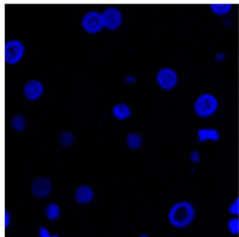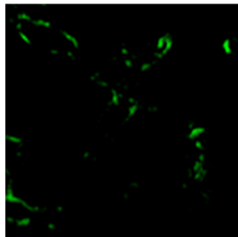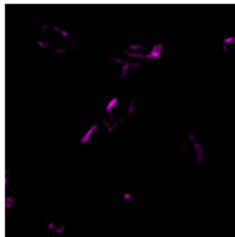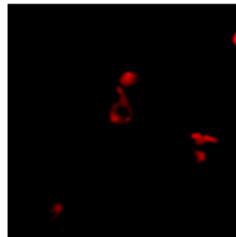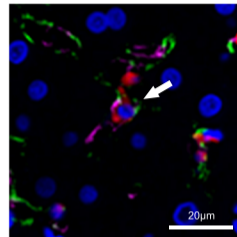

Additional S2/G1

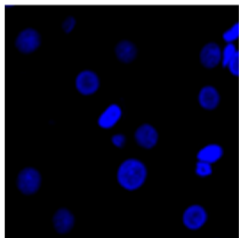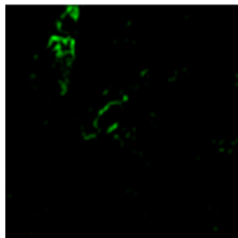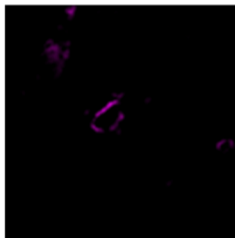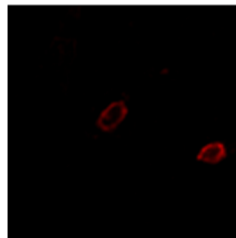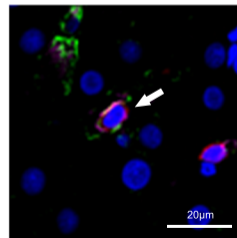

Supplement: Supplementary file 5 — Figure S5. Immunofluorescence results for additional two biopsies obtained from two G1 patients [file CTM2-12-e1073-s008.pdf]
