# Supplementary material for: Biopsy‐based single‐cell transcriptomics reveals MAIT cells as potential targets for controlling fibrosis‐related liver inflammation due to chronic hepatitis‐B infection
Source: Clin Transl Med. 2022 Oct 20;12(10):e1073. doi: 10.1002/ctm2.1073 (PMC9582669; doi:10.1002/ctm2.1073)
Supplement: Supplementary file 6 — Table S1. Clinical and biochemical parameters for chronic hepatitis B virus (HBV) patients [file CTM2-12-e1073-s005.docx]

**Table S1**. Clinical and biochemical parameters for chronic HBV patients

| Sample ID | Grade | Sex | Age | ALT (U/L) | ASL (U/L) | HBV-DNA  (log_10_ IU/ML) | Fibrosis score | No. cells |
| --- | --- | --- | --- | --- | --- | --- | --- | --- |
| S1 | G1 | female | 46 | 19 | 17 | 8.25 | 0 | 4984 |
| S2 | G2 | male | 44 | 32 | 24 | 3.68 | 1 | 5092 |
| S3 | G2 | female | 53 | 20 | 22 | LDL | 1 | 4716 |
| S4 | G2 | female | 55 | 210 | 118 | 6.66 | 1 | 3800 |
| S5 | G2 | male | 50 | 235 | 81 | 4.21 | 2 | 4038 |
| S6 | G2 | female | 60 | 41 | 33 | 4.53 | 2 | 1364 |
