# Supplementary material for: Biopsy‐based single‐cell transcriptomics reveals MAIT cells as potential targets for controlling fibrosis‐related liver inflammation due to chronic hepatitis‐B infection
Source: Clin Transl Med. 2022 Oct 20;12(10):e1073. doi: 10.1002/ctm2.1073 (PMC9582669; doi:10.1002/ctm2.1073)
Supplement: Supplementary file 7 — Table S2. Fraction of cells in each cell type for six samples. [file CTM2-12-e1073-s007.docx]

**Table S2**. Fraction of cells in each cell type for six samples.

|  | S1 | S2 | S3 | S4 | S5 | S6 | log2(FC) | *P* value |
| --- | --- | --- | --- | --- | --- | --- | --- | --- |
| T cell | 0.668 | 0.613 | 0.726 | 0.742 | 0.661 | 0.752 | 0.065 | < 0.01 |
| B cell | 0.037 | 0.055 | 0.050 | 0.048 | 0.045 | 0.050 | 0.423 | < 0.01 |
| NK-like cell | 0.086 | 0.089 | 0.048 | 0.029 | 0.053 | 0.067 | -0.588 | < 0.01 |
| cDC | 0.006 | 0.006 | 0.006 | 0.003 | 0.012 | 0.004 | 0.047 | < 0.01 |
| Macrophage | 0.095 | 0.155 | 0.080 | 0.087 | 0.131 | 0.047 | 0.074 | < 0.01 |
| HSC | 0.012 | 0.002 | 0.001 | 0.001 | 0.010 | 0.015 | -1.049 | < 0.01 |
| Hepatocyte | 0.030 | 0.009 | 0.007 | 0.016 | 0.028 | 0.006 | -1.184 | < 0.01 |
| Endothelial cell | 0.031 | 0.028 | 0.020 | 0.018 | 0.023 | 0.028 | -0.406 | < 0.01 |
| Cholangiocyte | 0.010 | 0.022 | 0.008 | 0.007 | 0.011 | 0.005 | 0.084 | < 0.01 |
| pDC | 0.004 | 0.009 | 0.003 | 0.003 | 0.009 | 0.001 | 0.322 | < 0.01 |
| 11 | 0.008 | 0.011 | 0.009 | 0.024 | 0.010 | 0.005 | 0.585 | < 0.01 |
| 12 | 0.012 | 0.002 | 0.042 | 0.021 | 0.005 | 0.020 | 0.561 | < 0.01 |
