# Supplementary material for: Biopsy‐based single‐cell transcriptomics reveals MAIT cells as potential targets for controlling fibrosis‐related liver inflammation due to chronic hepatitis‐B infection
Source: Clin Transl Med. 2022 Oct 20;12(10):e1073. doi: 10.1002/ctm2.1073 (PMC9582669; doi:10.1002/ctm2.1073)
Supplement: Supplementary file 8 — Table S3. Overview of T cell clusters and annotation [file CTM2-12-e1073-s009.docx]

**Table S3.** Overview of T cell clusters and annotation

| **Cluster names** | **Representative genes** | **Annotation** |
| --- | --- | --- |
| T0 | *PDCD1, TNFRSF9, CD27* | exhausted CD8+ T cell |
| T1 | *LTB, IL7R, CD52, TNFRSF4, AQP3* | circulating CD4^+^ T memory cell |
| T2 | *PIK3R1, CXCR4, CCL4, NR4A2* | activated CD8^+^ T cell |
| T3 | *TRDC, CMC1, GNLY, GZMB, PRF1, NKG7, KLRD1, CX3CR1* | cytotoxic γδT cell |
| T4 | *TRDC, CMC1, XCL1, XCL2* | activated γδT cell |
| T5 | *CX3CR1, FCGR3A, FGFBP2, GNLY, GZMB* | effector CD8^+^ T cell |
| T6, T7 | *RORC, ZBTB16, SLC4A10* | CD8^+^ mucosal-associated invariant T (MAIT) cell |
| T8 | *FTL* | immunosuppressive CD4^-^CD8^-^ T cell |
