# Supplementary material for: Biopsy‐based single‐cell transcriptomics reveals MAIT cells as potential targets for controlling fibrosis‐related liver inflammation due to chronic hepatitis‐B infection
Source: Clin Transl Med. 2022 Oct 20;12(10):e1073. doi: 10.1002/ctm2.1073 (PMC9582669; doi:10.1002/ctm2.1073)
Supplement: Supplementary file 9 — Supporting information1 [file CTM2-12-e1073-s004.docx]

**Connections between MAIT cells and other immune cells**

It is reported that MAIT cells helped coordinating the adaptive immune response^1^ by promoting inflammatory monocyte differentiate into dendritic cells^2^, matured monocyte-derived and primary DCs, which were to some extent consistent with the extensive cross-talk between MAIT cells and macrophage observed in this study (Figure 4b). Moreover, the ligand-receptor pairs such as ‘HLA-C_KIR2DL3’^3^, ‘HLA-A_KIR3DL1’^3^, ‘HLA-C_KIR2DL1’^3^, and ‘HLA-E_KLRK1’^3^ demonstrated extensive cross-talk for T7-NK cells and T7-T2, a population of activated CD8^+^ T lymphocytes. KIRs are a family of activating and inhibitory type I transmembrane glycoproteins with two to three extracellular domains expressed by NK cells and some T cell lymphocytes^4, 5^. KIRs serve as key regulators of natural killer cell function through their interactions with human leucocyte antigens (HLA), which will in turn function as helper masters to antiviral T cell responses^6^. Nowadays, various KIRs such as KIR2DL3 is reported to confer protection against HBV chronic infection^7^. KLRK1, also named as NKG2D, is a key activator of cytotoxic responses of T and NK cells. Combined with the upregulated pathways ‘Regulation of natural killer cell differentiation’ and ‘Regulation of natural killer cell activation’, T7 and T6 may assist virus infection control via activating NK cell and activated CD8^+^ T cell mediated immune responses. In addition, we observed crosstalk for T6-B cells and T7-B cells by ligand-receptor pair ‘PTPRC_CD22’, where PTPRC, also known as CD45, is an essential regulator of B cell antigen receptor-mediated activation^8^. Given the enriched pathway ‘B cell homeostasis’, T7 may help improve B-cell responses and promote the reactivation and differentiation of memory B cells^9^ in a more active way. Such results indicated that MAIT cell population T7 may have active antiviral functions for reducing HBV replication in grade G1 by cross-talking with other immune cells.

**References:**

[1] Salio M, Gasser O, Gonzalez-Lopez C, et al. Activation of Human Mucosal-Associated Invariant T Cells Induces CD40L-Dependent Maturation of Monocyte-Derived and Primary Dendritic Cells. *J Immunol* 2017; **199**(8): 2631-2638.

[2] Meierovics AI, Cowley SC. MAIT cells promote inflammatory monocyte differentiation into dendritic cells during pulmonary intracellular infection. *J Exp Med* 2016; **213**(12): 2793-2809.

[3] Shao X, Liao J, Li C, et al. CellTalkDB: a manually curated database of ligand-receptor interactions in humans and mice. *Brief Bioinform* 2021; **22**(4):bbaa269.

[4] Caligiuri MA. Human natural killer cells. *Blood* 2008; **112**(3): 461-469.

[5] Campbell KS, Purdy AK. Structure/function of human killer cell immunoglobulin-like receptors: lessons from polymorphisms, evolution, crystal structures and mutations. *Immunology* 2011; **132**(3): 315-325.

[6] Zheng M, Sun R, Wei H, et al. NK Cells Help Induce Anti-Hepatitis B Virus CD8+ T Cell Immunity in Mice. *J Immunol* 2016; **196**(10): 4122-4131.

[7] Di Bona D, Aiello A, Colomba C, et al. KIR2DL3 and the KIR ligand groups HLA-A-Bw4 and HLA-C2 predict the outcome of hepatitis B virus infection. *J Viral Hepat* 2017; **24**(9): 768-775.

[8] Al Barashdi MA, Ali A, McMullin MF, et al. Protein tyrosine phosphatase receptor type C (PTPRC or CD45). *J Clin Pathol* 2021; **74**(9): 548-552.

[9] Bennett MS, Trivedi S, Iyer AS, et al. Human mucosal-associated invariant T (MAIT) cells possess capacity for B cell help. *J Leukoc Biol* 2017; **102**(5): 1261-1269.
