# Supplementary material for: Biopsy‐based single‐cell transcriptomics reveals MAIT cells as potential targets for controlling fibrosis‐related liver inflammation due to chronic hepatitis‐B infection
Source: Clin Transl Med. 2022 Oct 20;12(10):e1073. doi: 10.1002/ctm2.1073 (PMC9582669; doi:10.1002/ctm2.1073)
Supplement: Supplementary file 10 — Supporting information1 [file CTM2-12-e1073-s002.docx]

**Materials and methods**

**1. Human samples and histological evaluation**

The study was conducted after obtaining ethical approval from the Ethics Committee of the first affiliated hospital of Zhejiang University (Zhejiang, China). Six patients with only chronic hepatitis B virus infection, whose HBsAg and HBV-DNA were positive, were enrolled at the first affiliated hospital of Zhejiang University from September 2019 to January 2020. Those patients with chronic liver disease related to ethanol abuse, NAFLD (nonalcoholic fatty liver disease), autoimmune liver diseases, and other common etiologies were excluded. Informed consent was obtained from each participant before enrollment. After receiving informed consent, liver biopsies were obtained from each patient for single cell sequencing analysis and histological evaluation of inflammation. For histopathological examination, sections were cut (5μm thick), stained with hematoxylin and eosin (H&E) and evaluated by an expert liver pathologist. The grade of inflammation was determined according to the Scheuer method^1^. Clinical and biochemical parameters including sex, age, serum aspartate aminotransferase (AST), alanine transaminase (ALT) and grades of inflammation are provided in Table S1. The H&E results for three representative patients are illustrated in Figure S1. Two formalin-fixed, paraffin-embedded liver biopsies from two additional G1 patients were involved to validate the differences between G1 and G2.

**2. Preparation of single-cell suspensions**

Core needle of liver biopsies were stored in tissue storage solution (Miltenyi, 130-100-008) on ice and were transferred for dissociation within minimum ischemic time. On arrival, each sample was washed in PBS with 0.04% BSA (Beyotime, ST023), and then was transferred to a gentleMACS C Tube (Miltenyi #130-093-237) containing 2.5 ml digestion enzyme mix prepared according to the kit user guide (Liver dissociation Kit from Miltenyi Biotech # 130-105-807). The C tube was then placed on a gentleMACS^TM^ Octo Dissociator (Miltenyi, 130-096-427) for mechanical dissociation using program 37C_m_LIDK_1. Upon run completion, the suspended cells were filtered using a 70 μm SmartStrainer (Miltenyi, 130-098-462). Red blood cells were lysed using red blood cell lysis solution (Miltenyi, 130-094-183). The remaining cell suspension was then centrifuged at 300xg and 4°C for 5 min. The resulting cell pellet was resuspended in 50-60μl PBS with 0.04% BSA to adjust cell concentration. During the dissociation procedures, cells were maintained on ice whenever possible, and the entire procedure was completed as fast as possible.

**3. Single-cell capture and cDNA library preparation**

After preparation of single-cell suspension, cell counts and viability was assessed via Trypan Blue (Thermo Fisher) using a hemocytometer (Thermo Fisher). The appropriate volume for each sample was calculated for a target capture of 10,000 cells. After GEM generation and barcoding followed by GEM RT reaction and cleanup steps, the obtained cDNA was purified and amplified for 12 cycles before being cleaned up using SPRlselect beads (Beckman). The cDNA concentration was then determined by a Bioanalyzer (Agilent Technologies). The cDNA libraries were prepared as recommended by the Single Cell 3^’^ Reagent Kits v2 user guide, and then cryopreserved at -20 °C until sequencing.

**4. Droplet-based single cell RNA sequencing and data processing**

The size of each library measured by a Bioanalyzer (Agilent Technologies) and qPCR amplification data (Kappa/Roche) was utilized to calculate the molarity of each library. In each run, libraries from 2 or 3 samples were pooled and normalized to a final loading concentration. After cluster generation using cBot (Illumina), samples were sequenced on a HiSeq 2500 with the following run parameters: read 1 - 26 cycles, read 2 – 98 cycles, index1 – 8 cycles. The target median sequencing depth is 60,000 reads per cell for each sample. Raw sequencing data (bcl file) were converted to fastq files with Illumina bcl2fastq (version 2.19.1). The CellRanger (10x Genomics) analysis pipeline including alignment, tagging, gene and transcript counting, and normalizing sequencing depth was then used to generate a digital gene expression matrix from this data. During demultiplex, only 1 mismatch in the barcodes was allowed. Genes detected in more than 0.1% of all the cells were retained. Cells with less than 500 detected genes and less than 700 UMI counts were excluded. Additionally, we removed doublets, filtered cells with the percentage of mitochondrial genes high than 20%^2, 3^. The total number of transcripts in each single cell was further normalized using scran^4^. Seurat package (version 4.0)^5-7^ on R (version 4.0.2) was utilized to detect highly variable genes, to scale the data, to cluster the cells, and to perform dimension reduction analyses such as PCA (principal component analysis) and UMAP (uniform manifold approximation and projection)^8^. The cell type annotation was conducted using scCATCH^9^.

**5. Subclusters for T cells and macrophage**

A shared nearest neighbor modularity optimization-based clustering algorithm implemented in *FindClusters* function of R Seurat package was utilized to conduct clustering of T cells and macrophage with parameters set as default. T cell subclusters were assigned as CD8^+^ and CD4^+^ according to marker genes from Xcell^10^. Differential gene expression analysis was further performed among different subclusters of T cells and macrophage, and each subcluster was annotated based on marker genes. Among CD8^+^ T cells, two populations of MAIT cells expressing marker genes RORC, ZBTB16 and SLC4A10 were identified as CD3^+^SLC4A10^+^TNFAIP3^+^ and CD3^+^SLC4A10^+^TNFAIP3^-^ T cells, respectively. The M1 and M2 signature score of Ma1, Ma2 and Ma3 cells were generated according to the expression of M1 (e.g., *TNF*, *CXCL9*, *CXCL10*, *IL12A*) and M2 (e.g., *TGFB1*, *CD163*, *CCL18*, *MRC1*) feature genes^11^. Both signature scores were defined as the average normalized expression of corresponding genes based on previous literature^12^.

**6. Immunofluorescence**

Sections of human liver biopsy samples were washed twice in PBST. After blocking, sections were incubated with anti-CD3 (Abcam, ab16669, 1:100), anti-SLC4A10 (Abcam, ab122229, 1:50), and anti-TNFAIP3 (Abcam, ab13597, 1:100) antibodies at 4℃ overnight. After washing, slides were treated with secondary antibodies Alexa Fluor 488 goat anti-mouse (Abcam, ab150113), Alexa Fluor 555 goat anti-rabbit (Abcam, ab150078), and Alexa Fluor 647 goat anti-rabbit (Abcam, ab150079). Fluorescent mounting medium with DAPI (ZSGB-BIO, ZLI-9557) was then added to the slides. Images were taken using a confocal microscope Lecia TCS SP8.

**7. Trajectory inference analysis**

Monocle2 (<http://cole-trapnell-lab.github.io/monocle-release>)^13^ was utilized to conduct trajectory inference analysis, where the pseudotime of single cells were calculated. The cells were reduced dimensionality by the DDRTree method, ordered along a trajectory and finally visualized^13^.

**8. Gene set enrichment analysis (GSEA)**

Pathway analysis was performed on hallmark gene sets in Molecular Signatures Database (MSigDB v7.1) using GSEA (version 4.1.0)^14^. We performed 100 permutations on genes differentially expressed between two MAIT cell populations selected by function *FindMarkers* in Seurat. A value of *P* < 0.05 and a false discovery rate (FDR) < 0.05 were considered statistically significant.

**9. Gene set variation analysis (GSVA)**

Gene set variation analysis (GSVA)^15^, an unsupervised gene set enrichment method that can estimate the scores for certain pathways or markers within a sample population^15^, was performed to evaluate the differential pathways between the two MAIT cell subclusters. We downloaded the ‘c5.all.v7.1.symbols.gmt’ gene sets from the Molecular Signatures Database for GSVA. The differential analysis of the gene sets was carried out using the limma package with R software. The gene sets with adjusted *P* < 0.05 was regarded as having differentially expressed genes.

**10. Cell-cell crosstalk analysis**

CellPhoneDB (version 2.1.1)^16^, a public repository of curated receptors, ligands and their interactions, was utilized to study the cell-cell crosstalk according to manufacturer’s manual (<https://www.cellphonedb.org/>). The mean value, calculated based on the percentage of cells expressing the specific gene and the gene expression mean, represents the average ligand and receptor expression in a specific cell type. The *P*-value, representing the likelihood of a specific cell type of a given receptor-ligand complex, is calculated based on the proportion of the means that are as high or higher than the actual mean.

**11. External validation analysis**

We re-utilized the single-cell RNA sequencing data recently published by Zhang *et al.* in Gut^17^, which provided the single-cell RNA sequencing data generated from paired liver and blood samples obtained from 17 HBV-infected patients and 6 HBV-free healthy controls (HCs). By filtering samples with the number of single cells fewer than 4000, we selected the single-cell RNA sequencing data generated from liver samples obtained from 4 HBV-infected patients with grade G1 and 4 HBV-infected patients with grade G2. Firstly, we retrieved a population of CD8^+^ T cells from the external validation dataset by selecting cells that expressed genes *CD3D* and *CD8A* to confirm the decrease of MAIT cells in grade G2 as compared to G1. Then, a population of CD8^+^ MAIT cells were obtained by selecting cells that expressed genes *CD3D*, *CD8A* and *SLC4A10* at the same time according to *Zhang*’s study.^17^ Based on the findings of our study that the expression of gene *TNFAIP3* differed between two MAIT cell subpopulations (T7 and T6), we further divided the population of CD8^+^ MAIT cells into two subpopulations according to the expression of gene *TNFAIP3* to validate the main findings that two subpopulations of MAIT cells were present in the CD8^+^ MAIT cells in both grades G1 and G2, and that T7 dominated in grade G1 but the proportion of T7 in all CD8^+^ MAIT cells decreased in grade G2 as compared to G1. Moreover, the ratio of the cell number proportions between T6 and T7 was also involved to confirm the increase of T6 in CD8^+^ MAIT cells during the progression from grade G1 to G2.

**12. Statistical analysis and data visualization**

Cell populations associated with liver inflammation progression were evaluated based on fold change (FC >2.5) and *P* value of Chi-square test (*P* < 0.01) by comparing cell number proportions between grades G1 and G2. All statistical analyses and data visualization in this study were performed with R software (version 4.0.2).

**References:**

[1] Scheuer PJ. Classification of chronic viral hepatitis: a need for reassessment**.** *J Hepatol.* 1991;**13**(3):372-374.

[2] Vento-Tormo R, Efremova M, Botting RA, et al. Single-cell reconstruction of the early maternal-fetal interface in humans**.** *Nature.* 2018;**563**(7731):347-353.

[3] Ma L, Hernandez MO, Zhao Y, et al. Tumor Cell Biodiversity Drives Microenvironmental Reprogramming in Liver Cancer**.** *Cancer Cell.* 2019;**36**(4):418-430.

[4] Lun AT, Bach K, Marioni JC. Pooling across cells to normalize single-cell RNA sequencing data with many zero counts**.** *Genome biology.* 2016;**17**:75.

[5] Stuart T, Butler A, Hoffman P, et al. Comprehensive Integration of Single-Cell Data**.** *Cell.* 2019;**177**(7):1888-1902.

[6] Butler A, Hoffman P, Smibert P, et al. Integrating single-cell transcriptomic data across different conditions, technologies, and species**.** *Nat Biotechnol.* 2018;**36**(5):411-420.

[7] Satija R, Farrell JA, Gennert D, et al. Spatial reconstruction of single-cell gene expression data**.** *Nat Biotechnol.* 2015;**33**(5):495-502.

[8] Becht E, McInnes L, Healy J, et al. Dimensionality reduction for visualizing single-cell data using UMAP**.** *Nat Biotechnol.* 2019;**37**:38-44.

[9] Shao X, Liao J, Lu X, et al. scCATCH: Automatic Annotation on Cell Types of Clusters from Single-Cell RNA Sequencing Data**.** *iScience.* 2020;**23**(3):100882.

[10] Aran D, Hu Z, Butte AJ. xCell: digitally portraying the tissue cellular heterogeneity landscape**.** *Genome Biol.* 2017;**18**(1):220.

[11] He D, Wang D, Lu P, et al. Single-cell RNA sequencing reveals heterogeneous tumor and immune cell populations in early-stage lung adenocarcinomas harboring EGFR mutations**.** *Oncogene.* 2021;**40**(2):355-368.

[12] Xie X, Shi Q, Wu P, et al. Single-cell transcriptome profiling reveals neutrophil heterogeneity in homeostasis and infection**.** *Nat Immunol.* 2020;**21**(9):1119-1133.

[13] Trapnell C, Cacchiarelli D, Grimsby J, et al. The dynamics and regulators of cell fate decisions are revealed by pseudotemporal ordering of single cells**.** *Nat Biotechnol.* 2014;**32**(4):381-386.

[14] Subramanian A, Tamayo P, Mootha VK, et al. Gene set enrichment analysis: a knowledge-based approach for interpreting genome-wide expression profiles**.** *Proc Natl Acad Sci U S A.* 2005;**102**(43):15545-15550.

[15] Hanzelmann S, Castelo R, Guinney J. GSVA: gene set variation analysis for microarray and RNA-seq data**.** *BMC Bioinformatics.* 2013;**14**:7.

[16] Efremova M, Vento-Tormo M, Teichmann SA, et al. CellPhoneDB: inferring cell-cell communication from combined expression of multi-subunit ligand-receptor complexes**.** *Nat Protoc.* 2020;**15**(4):1484-1506.

[17] Zhang C, Li J, Cheng Y, et al. Single-cell RNA sequencing reveals intrahepatic and peripheral immune characteristics related to disease phases in HBV-infected patients**.** *Gut.* 2022;**0**:1-15.
